# Neurofeedback for Binge‐Eating Disorder: Neurophysiological Outcome Predictors and Rapid Response

**DOI:** 10.1002/eat.70023

**Published:** 2026-01-07

**Authors:** Ben Schreglmann, Ricarda Schmidt, Michael Lührs, Anja Hilbert

**Affiliations:** ^1^ Integrated Research and Treatment Center Adiposity Diseases, Behavioral Medicine Research Unit, Department of Psychosomatic Medicine and Psychotherapy Leipzig University Medical Center Leipzig Germany; ^2^ Faculty of Psychology and Neuroscience Maastricht University Maastricht the Netherlands; ^3^ Brain Innovation B.V. Maastricht the Netherlands

**Keywords:** binge‐eating disorder, brain‐directed treatments, EEG, electroencephalography, fNIRS, functional near‐infrared spectroscopy, neurofeedback, neurophysiological markers, outcome predictors, rapid response

## Abstract

**Objective:**

Pioneer studies suggested the effectiveness of food‐specific electroencephalography (EEG) and real‐time functional near‐infrared spectroscopy (rtfNIRS) neurofeedback (NF) trainings in the treatment of binge‐eating disorder (BED). These trainings aim to improve participants' neurophysiological self‐regulation. However, pretreatment neurophysiological activity, a supposed key predictor of NF outcomes, remains unexplored.

**Method:**

This preregistered analysis (https://osf.io/xsrj3) used data from a randomized‐controlled trial (DRKS00014752) on 47 adults (47 ± 13 years, 81% women) with interview‐assessed BED having undergone food‐specific EEG‐ or rtfNIRS‐NF (12 sessions, over 8 weeks). Bayesian linear models explored pretreatment fronto‐central high beta power (23–28 Hz; indicating increased attention) in EEG, pretreatment prefrontal oxygenation in fNIRS (indicating increased cognitive control), and rapid response (RR; early reductions in binge eating) as predictors of treatment outcomes reflecting mental and physical health at posttreatment and 6‐month follow‐up.

**Results:**

Higher high beta power during passive viewing of food pictures, lower high beta power, and less oxygenation during regulatory NF tasks, as well as RR predicted more favorable primary outcomes, including reduced objective binge‐eating episodes and increased abstinence from binge eating. Overall, neurophysiological predictors—especially EEG activity—showed greater predictive value than RR.

**Conclusion:**

The preliminary findings suggest the relevance of neurophysiological activity in the prediction of NF treatment outcomes in BED. While patients with increased involuntary attention in response to food stimuli profited most from EEG‐NF, those with greater difficulties in voluntary recruitment of food‐related cognitive control profited most from rtfNIRS‐NF. The predictors identified could guide future treatment allocation and represent a first step toward more individualized therapy approaches.

## Key Points

- Pretreatment EEG and fNIRS activity predicted outcomes of neurofeedback for binge‐eating disorder with a larger effect size than rapid response, the currently most relevant predictor for binge‐eating disorder therapy outcomes.
- Patients with stronger involuntary EEG response to food stimuli and better voluntary EEG regulation benefited more from EEG neurofeedback.
- Patients with worse voluntary fNIRS regulation benefited more from fNIRS neurofeedback.

## 1 Introduction

Binge‐eating disorder (BED), characterized by recurrent objective binge‐eating episodes (OBEs) without regular compensatory behavior (American Psychiatric Association 2022), is the most prevalent eating disorder with a global pooled point/12‐month prevalence of 0.9% (Erskine and Whiteford 2018; Galmiche et al. 2019). BED is associated with numerous mental and somatic comorbidities (Hambleton et al. 2022; Keski‐Rahkonen 2021), increased standardized mortality (Fichter and Quadflieg 2016), and significant social and economic costs (Ling et al. 2017; Streatfeild et al. 2021).

When confronted with food stimuli, individuals with BED show increased fronto‐central high beta power in electroencephalography (EEG), indicating heightened food‐related attention, and prefrontal hypoactivation in functional near‐infrared spectroscopy (fNIRS), indicating reduced food‐related cognitive control (Blume et al. 2019; Hilbert et al. 2024; Rösch et al. 2021). While cognitive‐behavioral therapy—the current treatment of choice for BED (Hilbert et al. 2019)—does not directly target these neurophysiological factors, brain directed treatments such as EEG‐ and real‐time fNIRS (rtfNIRS) neurofeedback (NF) do (Hilbert et al. 2024). EEG and fNIRS are non‐invasive techniques measuring electrical brain activity and cortical blood oxygenation, respectively. Through real‐time visual feedback on individual EEG or fNIRS signals, NF enables patients to develop self‐regulation strategies (Hilbert et al. 2024; Wimmer et al. 2023). Specifically, EEG‐NF trains the downregulation of fronto‐central high beta power to improve attention regulation, whereas rtfNIRS‐NF trains the upregulation of prefrontal oxygenation to enhance cognitive control in relation to food (Blume et al. 2022; Hilbert et al. 2024). This may underly reductions in OBE frequency following EEG‐ and rtfNIRS‐NF in patients with BED (Blume et al. 2022; Hilbert et al. 2024). Possibly also enhancing self‐efficacy and perceived control, NF could represent a promising adjunct to psychotherapy (Bartholdy et al. 2013). Nevertheless, initial evidence suggests that standalone NF for BED achieves full remission in only 23.0%–36.8% of patients posttreatment (Blume et al. 2022; Hilbert et al. 2024)—compared to ~50% in standalone cognitive‐behavioral therapy (Linardon et al. 2018)—highlighting the need to identify patients benefiting most. Lower baseline OBE frequency, lower baseline food cravings, lower baseline body mass index (BMI, kg/m^2^), and older age were already found to predict lower OBE frequency posttreatment and at 6‐month follow‐up in patients with BED undergoing EEG‐ or rtfNIRS‐NF (Rösch, Schmidt, and Hilbert 2023). However, all these predictors were small‐ or less than small‐sized.

Regarding NF outcome predictors in general, a systematic review (Weber et al. 2020) identified pretreatment neurophysiological activity—particularly parameters targeted during the training—as most reliable. In EEG‐NF trainings, pretreatment power in the targeted frequency bands predicted symptom reductions in children with attention‐deficit/hyperactivity disorder (*n* = 46; Gevensleben et al. 2009), adults with obsessive‐compulsive disorder (*n* = 15; Kopřivová et al. 2013), and adults with nicotine addiction (*N* = 60; Meng et al. 2023). Overall, higher pretreatment EEG activity in targeted frequency bands seemed to predict better outcomes when EEG activity upregulation was the goal, whereas evidence for downregulation protocols was mixed and limited (Weber et al. 2020). Regarding pretreatment fNIRS activity, lower prefrontal oxygenation during rest or cognitive tasks predicted symptom reductions in pharmacological and neurostimulation therapies for major depressive disorder (Ho et al. 2020). Furthermore, fNIRS activity has been theorized to predict outcomes of behavioral treatments in addiction (Bunce et al. 2013), as well as pharmacological and NF treatments in schizophrenia (Koike et al. 2013). Nevertheless, to the authors' knowledge, pretreatment fNIRS activity has not yet been used to predict NF outcomes and neurophysiological activity has not yet been used to predict clinical outcomes for eating disorders, including BED.

In this context, based on a randomized‐controlled trial comparing food‐specific high‐beta EEG‐NF and individualized food‐specific rtfNIRS‐NF for BED, the present study explored pretreatment EEG and fNIRS activity as predictors of OBE frequency, abstinence from binge eating, and secondary outcomes of mental and physical health at posttreatment and 6‐month follow‐up. For comparison, rapid response (RR)—an early reduction in OBE frequency, typically within the first four weeks of treatment—was explored as a predictor of these outcomes, as it represents the most consistent predictor of psychological and medical therapy outcomes for BED (Vall and Wade 2015).

## 2 Materials and Methods

### 2.1 Design

This study is a secondary analysis of data from a single‐center, assessor‐blinded, randomized‐controlled feasibility trial. A more detailed description of the design was given before (Hilbert et al. 2024). Briefly, participants were randomized to food‐specific EEG‐NF (*n* = 25), rtfNIRS‐NF (*n* = 28), or waitlist (*n* = 25). The waitlist group was offered rtfNIRS‐NF after an 8‐week waiting period. Assessments were conducted at baseline (t0), pretreatment during the first NF session, posttreatment (t1), and at 6‐month follow‐up (t2). All study procedures were approved by the Ethics Committee of Leipzig University (05/03/2018; 476/17‐ek) and registered in the German Clinical Trials Register (DRKS00014752).

### 2.2 Participants

Participants were recruited at Leipzig University Medical Center between 06/2018 and 03/2020 through advertisements or clinic referrals and were offered €80 for assessment completion. *N* = 78 adults (age ≥ 18 years) provided written informed consent and met inclusion criteria of a diagnosis of BED or BED of low frequency and/or limited duration according to the *Diagnostic and Statistical Manual of Mental Disorders 5* (American Psychiatric Association 2022), a BMI between 25.0 and 44.9 kg/m^2^, sufficient German language skills, and a feasible commute to the research unit. Exclusion criteria comprised: severe mental (e.g., substance use disorder) or somatic (e.g., head injury) disorders; medications affecting eating behavior or weight (e.g., antipsychotics); hearing, vision, or language impairments with effect on testing; previous or planned bariatric surgery; ongoing psychotherapy; and current lactation or pregnancy.

Three participants from the rtfNIRS‐NF group were excluded due to fNIRS programming problems (Hilbert et al. 2024). In accordance with Rösch, Schmidt, and Hilbert (2023), the waitlist group was completely excluded from analyses due to an unexpectedly high spontaneous remission raising concerns about comparability (Hilbert et al. 2024). One participant from the EEG‐NF group and two participants from the rtfNIRS‐NF group were unable to start the intervention because of the COVID‐19 pandemic, resulting in an intent‐to‐treat sample of *N* = 47 (EEG‐NF *n* = 24, rtfNIRS‐NF *n* = 23). Of these participants, one from the EEG‐NF group and three from the rtfNIRS‐NF group were lost (not reached) at posttreatment, as were two EEG‐NF participants and one rtfNIRS‐NF participant at follow‐up. For more detailed information see the CONSORT flow chart (Hilbert et al. 2024).

### 2.3 Treatment

Participants were offered 12 NF sessions of 60 min each over an 8‐week period. NF was augmented by homework assignments grounded in cognitive‐behavioral principles, supporting transfer of NF skills to daily life. Prior to the first and the seventh training session, participants rated *n* = 70 food images (Blechert et al. 2014) regarding their relevance to OBEs and associated cravings. The 12 images most closely linked to recent binge eating and/or inducing the highest cravings were used as training stimuli.

The EEG‐NF built on a validated protocol (Hilbert et al. 2019) with adaptations including downregulation of high beta power (23–28 Hz). Each session consisted of: Two 180 s resting‐state baseline periods; 12 regulation trials of 60 s each, tasking participants to reduce their high beta power and muscle activity below their established baseline, represented by two dynamic bars on the screen; and six transfer trials of 60 s each, tasking participants to reduce their high beta power without real‐time feedback. Before each regulation and transfer trial, one individualized food image was presented for 25 s, prompting participants to vividly imagine the food displayed. Each session started with a baseline period, continued with regulation trials, another baseline period, and ended with transfer trials.

The rtfNIRS‐NF built on prior real‐time magnetic resonance imaging NF research (Ihssen et al. 2017; Sokunbi et al. 2014). Using a food‐related Go/NoGo task as functional localizer, two adjacent channels in individual brain regions of interest (ROIs) in the dorsolateral prefrontal cortex or the inferior frontal gyrus were selected for training. Each session consisted of: 12 regulation trials of 30s each, tasking participants to upregulate their oxyhemoglobin levels in the ROIs, represented by the size of a food image; 12 mirror trials of 30s each presenting food images with static size; and 12 transfer trials of 30s each, tasking participants to upregulate their oxyhemoglobin levels, while visual feedback was provided only during the last 2 s. Image size decreased when oxyhemoglobin levels exceeded the previous sampling point. Each session started with alternating regulation and mirror trials and ended with transfer trials.

## 3 Measures

Table S1 provides an overview of all measures and respective computations.

### 3.1 Outcomes

Primary outcomes OBE frequency and abstinence from binge eating (defined as an OBE frequency of zero) over the past 28 days were assessed at posttreatment and follow‐up with the Eating Disorder Examination (EDE; (Hilbert and Tuschen‐Caffier 2016a)) by trained interviewers blind to randomization.

Secondary outcomes, assessed via patient‐report questionnaires at posttreatment and follow‐up, included eating disorder psychopathology (Eating Disorder Examination‐Questionnaire [EDE‐Q]; Hilbert and Tuschen‐Caffier 2016b), food cravings (Food Cravings Questionnaire‐Trait‐Reduced [FCQ‐T‐r]; Meule et al. 2014), depressive symptoms (Patient Health Questionnaire Depression Scale [PHQ‐9]; Löwe et al. 2004), anxiety symptoms (Generalized Anxiety Disorder Scale [GAD‐7]; Löwe et al. 2008), and quality of life (Short Form Health Survey [SF‐12]; Gandek et al. 1998). BMI and waist‐to‐hip ratio were objectively measured at posttreatment and follow‐up. Higher scores indicated higher impairment except for the SF‐12, where higher scores indicated lower impairment. Internal consistencies (*ω* = 0.75–0.94) and the code used for score calculations have been reported previously (Hilbert et al. 2024).

### 3.2 Predictors

Since neurophysiological activity was not assessed at baseline, data from the first training session (pretreatment) were used for prediction. In both protocols, neurophysiological activity during passive‐viewing trials was interpreted as involuntary activity, whereas activity during the active regulation and transfer trials was interpreted as voluntary. Data recording and preprocessing are detailed in the Supporting Information.

In EEG‐NF, fronto‐central high beta power (23–28 Hz; μV^2^) was recorded during a passive‐viewing food‐cue presentation displaying three individualized food images for 30s each, as well as throughout the regulation and transfer trials. For each participant, three EEG predictors of therapy outcomes were calculated, corresponding to mean fronto‐central high beta power during passive‐viewing, regulation, and transfer trials.

In rtfNIRS‐NF, prefrontal fNIRS signals of the two adjacent channels in the individual ROIs were recorded throughout mirror (passive‐viewing), regulation, and transfer trials. Trial‐specific generalized linear models were applied to the preprocessed data in Satori version 2.0.6 (NIRx Medical Technologies 2024), with predictor time courses obtained by convolving a condition boxcar time course with a standard two‐gamma hemodynamic response function. The resulting generalized linear models' beta coefficients (*β*) were interpreted as trial‐wise changes in oxyhemoglobin levels—with positive values indicating increasing and negative values indicating decreasing oxyhemoglobin levels—and averaged between the two adjacent channels. For each participant and trial type, the percentage of trials showing oxygenation (*β* > 0.2) and deoxygenation (*β* < −0.2) was calculated relative to the total number of trials of that type. These percentages yielded six fNIRS predictors of therapy outcomes corresponding to oxygenation and deoxygenation during passive‐viewing, regulation, and transfer trials.

For comparison, RR was explored as a predictor. A Receiver Operating Characteristics Analysis (see Supporting Information) yielded a reduction in OBE frequency of ≥ 91.67% at week 4 of treatment as most predictive for posttreatment abstinence from binge eating. This definition was compared to the more frequently used definition by Grilo et al. (2006), a reduction of ≥ 65% by week 4 of treatment and showed similar or better predictive performance in all models. Therefore, only results based on the 91.67% cutoff were reported. As preregistered (https://osf.io/xsrj3), baseline and pretreatment predictors of RR were also explored. Corresponding analyses are detailed in the Tables S9 and S11.

### 3.3 Sociodemographics and Covariates

Sociodemographics, assessed via patient‐report at baseline, included sex, age, years of school education, professional situation, and illness duration. To control for baseline differences, baseline values of all outcomes were included as covariates.

### 3.4 Missing Data

Since the missing data pattern did not significantly deviate from randomness (Little 1988), χ^2^(103) = 107.00, *p* = 0.37, missing values were imputed using Multiple Imputation by Chained Equations (van Buuren and Groothuis‐Oudshoorn 2011) in R version 4.4.1 (R Core Team 2024), employing predictive mean matching (5 imputations, 10 iterations each, seed = 42). Results were pooled for final analysis. EEG and fNIRS data were not imputed due to their individual character. Table S1 provides detailed numbers of imputed values.

### 3.5 Data Analysis

#### 3.5.1 Sample Descriptives

Baseline or pretreatment values of all measures were reported descriptively. Supplementary correlations were computed between all baseline measures and pretreatment neurophysiological measures, as well as between change in neurophysiological activity first versus last attended NF session and posttreatment abstinence from binge eating. Correlations were interpreted according to Cohen (1988); small, *ρ* ≥ 0.10; medium, *ρ* ≥ 0.30; large, *ρ* ≥ 0.50.

#### 3.5.2 Linear Models

Predictive analyses were preregistered after data collection but prior to analysis (https://osf.io/xsrj3). To maintain clarity and conciseness, this manuscript focuses on pretreatment neurophysiological predictors of primary therapy outcomes, including OBE frequency and abstinence from binge eating, using RR as comparison. Additional preregistered analyses, not central to the manuscript's primary aims, including prediction of secondary outcomes and the prediction of RR using baseline and pretreatment variables, are fully detailed in the Supporting Information.

Given that frequentist null‐hypothesis significance testing has been shown to lead to serious issues with the reproducibility and reliability of scientific results (Benjamin et al. 2018; Colquhoun 2017, 2019), Bayesian adaptations of linear models (Gelman et al. 2013) were employed in R version 4.4.1 (R Core Team 2024) using the *brms* package (Bürkner 2017) to assess predictors of therapy outcomes. Table S2 provides a structured overview of employed models. To address collinearity, predictors showing an (adjusted) Generalized Variance Inflation Factor > 5 were removed (Hair et al. 2013), starting with the highest value. To prevent overfitting, a backward elimination approach was employed: Predictors whose 95% credible interval for the regression parameter included 0 were excluded stepwise, starting with the regression parameter whose mean value was closest to 0. Covariates were never excluded. Model performance was assessed via Leave‐One‐Out Cross‐Validation expected log predictive density (Vehtari et al. 2017); if a decrease > 4 after an exclusion indicated worse predictive accuracy (Sivula et al. 2020), predictors were reinstated. Where applicable, metric predictors and outcomes were z‐standardized (*M* = 0, SD = 1). Ordinal and dichotomous predictors were effect coded (−0.5, 0.5), with cumulative coding for ordinal variables. Models were run with 8000 iterations, 4000 burn‐in samples, 8 chains, a target Metropolis acceptance probability of 0.8, a maximum tree depth of 15, and seed = 42. To assess model convergence and evaluate model fit, trace plots, autocorrelation plots, and visual posterior predictive checks were employed. Further, it was ensured that effective sample sizes were ≥ 1000 and Gelman‐Rubin statistics ($\hat{R}$) were < 1.1 (Gelman et al. 2013) by either pooling imputations using the *brms* combine_models() function or—if $\hat{R}$≥1.1—pooling them manually using Rubin's Rule (Rubin 1987). Corresponding R code is provided in the Supporting Information.

A predictor was considered influential if the 95% credible interval of its regression parameter did not include 0 (Gelman et al. 2013) in the main analysis and both sensitivity analyses (see below). As a measure of effect size, Bayesian *R* ^2^ and incremental Bayesian *R* ^2^ (Δ*R* ^2^) were calculated (Gelman et al. 2019). As Bayesian statistics treat variables as distributions rather than fixed values, no established interpretation cutoffs exist for Bayesian *R* ^2^. Nevertheless, referring to each distribution's mean value, effect size interpretations based on Cohen (1988); small, *R* ^2^ ≥ 0.02, medium, ≥ 0.13, large, ≥ 0.26 were reported as orientation.

#### 3.5.3 Priors

Normal distributions were employed as priors for all model parameters except for the dispersion parameter in the negative binomial regression and the precision parameter in the beta regression, where log‐normal distributions were employed. Given the exploratory nature of the analyses, an empirical Bayes approach was pursued, allowing the data to inform the priors by estimating hyperparameters from frequentist models (Efron and Morris 1973; Gelman et al. 2013). Specifically, maximum likelihood estimates from frequentist models served as estimates for hyperparameters of the corresponding prior distributions, while a common prior scale for each outcome type, model type, and predictor family (EEG, fNIRS, RR) was estimated to address multiplicity (Bishop 2006; Gelman et al. 2012). To examine the robustness of the results, sensitivity analyses using doubled and halved standard deviations for the priors were employed.

## 4 Results

### 4.1 Sample Descriptives

The sample was predominantly female (*n* = 38, 80.85%), had a mean age of 47.10 years (SD = 13.27, range = 22–78), and a mean BMI of 36.68 kg/m^2^ (SD = 5.07, range 26.57–44.88). Further sample descriptives are presented in Tables 1 and S10. Supplementary correlations are presented in Tables S3–S5.

**TABLE 1:** Baseline and pretreatment sample descriptives.

Construct	*M* (SD) or *n* (%)		Valid values (*n*)		Imputed values (*n*)	
	EEG‐NF	fNIRS‐NF	EEG‐NF	fNIRS‐NF	EEG‐NF	fNIRS‐NF
*Sociodemographics* ^a^						
Age (years)	46.33 (14.23)	47.91 (12.20)	24	23	0	0
Sex (female)	19 (79.17)	19 (82.61)	24	23	0	0
Education (≥ 12 years)	13 (54.17)	12 (52.17)	24	23	0	0
Professional situation						
Working full‐time	7 (29.17)	15 (65.22)	24	23	0	0
Working part‐time	7 (29.17)	2 (8.70)	24	23	0	0
Occasionally employed	1 (4.17)	3 (13.04)	24	23	0	0
Not working	9 (37.50)	3 (13.04)	24	23	0	0
*Anthropometrics* ^a^						
Body mass index (kg/m^2^)	36.77 (5.11)	36.59 (5.04)	24	23	0	0
Waist‐to‐hip ratio	0.88 (0.10)	0.89 (0.07)	24	22	0	1
*Eating‐disorder related variables* ^a^						
Illness duration (years)	10.45 (11.24)	10.25 (12.96)	21	22	0	0
OBE frequency (past 28 days; EDE)	12.08 (8.36)	13.91 (12.30)	24	23	0	0
Eating disorder psychopathology (EDE‐Q, 0–6)	2.97 (1.06)	2.70 (0.80)	23	22	1	1
Food cravings (FCQ‐T‐r, 15–75)	60.12 (12.48)	56.51 (11.58)	23	21	1	2
*General psychopathology* ^a^						
Depressive symptoms (PHQ‐9, 0–27)	8.86 (4.76)	9.26 (3.23)	23	22	1	1
Anxiety symptoms (GAD‐7, 0–21)	6.73 (4.75)	5.88 (3.41)	23	22	1	1
Mental quality of life (SF‐12)	42.87 (11.79)	42.24 (9.55)	23	22	1	1
Physical quality of life (SF‐12)	43.94 (10.51)	42.20 (8.58)	23	22	1	1
*EEG activity (fronto‐central high beta power)* ^b^						
Passive‐viewing trials (μV^2^)	6.08 (2.25)	—	22	—	0	—
Regulation trials (μV^2^)	4.91 (1.28)	—	21	—	0	—
Transfer trials (μV^2^)	5.21 (1.50)	—	21	—	0	—
*fNIRS activity (prefrontal oxyhemoglobin)* ^b^						
Passive‐viewing trials						
Oxygenation trials (%)	—	43.56 (27.05)	—	22	—	0
Deoxygenation trials (%)	—	29.17 (25.59)	—	22	—	0
Regulation trials						
Oxygenation trials (%)	—	28.03 (27.71)	—	22	—	0
Deoxygenation trials (%)	—	44.70 (33.63)	—	22	—	0
Transfer trials						
Oxygenation trials (%)	—	38.33 (30.59)	—	20	—	0
Deoxygenation trials (%)	—	32.92 (26.81)	—	20	—	0

Abbreviations: deoxygenation = percent of trials with *β* < −0.2; EDE = eating disorder examination (Hilbert and Tuschen‐Caffier 2016a); EDE‐Q = eating disorder examination‐questionnaire (Hilbert and Tuschen‐Caffier 2016b); FCQ‐T‐r = food cravings questionnaire‐trait reduced (Meule et al. 2014); GAD‐7 = generalized anxiety disorder 7 (Löwe et al. 2008); high beta power = 23–28 Hz; OBE = objective binge‐eating episode; oxygenation = percent of trials with *β* > 0.2; PHQ‐9 = patient health questionnaire depression scale (Löwe et al. 2004); SF‐12 = short form health survey (Gandek et al. 1998); *β* = regression parameter in the applied general linear models representing the change in oxyhemoglobin levels.

^a^ Assessed at baseline (t0).

^b^ Assessed pretreatment during first training session.

### 4.2 Linear Models

Following predictors showed robust associations with outcomes throughout the sensitivity analyses. Since irrelevant to the research question, effects of covariates were not reported in the text. However, detailed results of all linear models are presented in Table 2 (primary outcomes), as well as Tables S6–S8 (secondary outcomes) and S11 (predictors of RR).

**TABLE 2:** Results of the Bayesian linear models assessing pretreatment EEG activity and pretreatment fNIRS activity as predictors of primary outcomes.

Model and predictors	Regression coefficient (*β*)				Bayesian *R* ^2^ or Δ*R* ^2^			
	*M*	SD	95% CrI		*M*	SD	95% CrI	
			Lower	Upper			Lower	Upper
*EEG activity models (n = 19)*								
OBE frequency (EDE) posttreatment					0.32	0.19	0.04	0.67
Intercept	1.36	0.17	1.02	1.71				
OBE frequency (EDE) baseline^b^	0.33	0.18	−0.01	0.69	0.16	0.21	< 0.01	0.57
Passive‐viewing high beta power (EEG)^a^	−0.50	0.21	−0.91	−0.09	0.12	0.24	< 0.01	0.57
OBE frequency (EDE) follow‐up					0.48	0.08	0.33	0.63
Intercept	1.22	0.27	0.68	1.75				
OBE frequency (EDE) baseline^b^	0.52	0.28	−0.02	1.07	0.12	0.18	< 0.01	0.47
Passive‐viewing high beta power (EEG)	−1.59	0.47	−2.52	−0.67	0.26	0.23	< 0.01	0.70
Regulation high beta power (EEG)	0.80	0.29	0.22	1.37	0.14	0.22	< 0.01	0.58
Abstinence from binge eating (EDE) posttreatment					0.23	0.08	0.06	0.35
Intercept	−1.39	0.46	−2.32	−0.51				
OBE frequency (EDE) baseline	−0.22	0.53	−1.27	0.81	0.01	0.11	< 0.01	0.23
Passive‐viewing high beta power (EEG)	1.23	0.49	0.31	2.21	0.15	0.09	< 0.01	0.31
Transfer high beta power (EEG)^a^	−1.15	0.60	−2.34	−0.01	0.08	0.11	< 0.01	0.28
Abstinence from binge eating (EDE) follow‐up					0.36	0.05	0.23	0.43
Intercept	−0.36	0.44	−1.23	0.48				
OBE frequency (EDE) baseline	−1.11	0.63	−2.38	0.08	0.05	0.08	< 0.01	0.24
Passive‐viewing high beta power (EEG)	2.27	0.74	0.91	3.81	0.26	0.08	0.09	0.39
Transfer high beta power (EEG)	−1.28	0.58	−2.45	−0.17	0.10	0.08	< 0.01	0.28
*fNIRS activity models (n = 20)*								
OBE frequency (EDE) posttreatment					0.51	0.10	0.23	0.60
Intercept	1.47	0.16	1.15	1.80				
OBE frequency (EDE) baseline	0.88	0.18	0.53	1.25	0.45	0.12	0.14	0.59
Passive‐viewing deoxygenation (fNIRS)^b^	−0.25	0.18	−0.61	0.10	< 0.01	0.14	< 0.01	0.31
OBE frequency (EDE) follow‐up					0.33	0.22	< 0.01	0.77
Intercept	1.46	0.34	0.80	2.12				
OBE frequency (EDE) baseline	0.42	0.31	−0.20	1.04	0.23	0.27	< 0.01	0.76
Passive‐viewing oxygenation (fNIRS)	−0.36	0.29	−0.93	0.20	0.09	0.34	< 0.01	0.77
Abstinence from binge eating (EDE) posttreatment					0.53	0.09	0.31	0.66
Intercept	−6.42	1.72	−9.95	−3.22				
OBE frequency (EDE) baseline	−7.76	2.17	−12.19	−3.66	0.42	0.11	0.17	0.61
Passive‐viewing oxygenation (fNIRS)^a^	1.56	0.79	0.02	3.15	0.08	0.13	< 0.01	0.37
Transfer deoxygenation (fNIRS)	2.63	0.96	0.84	4.57	0.23	0.14	< 0.01	0.51
Abstinence from binge eating (EDE) follow‐up					0.25	0.07	0.12	0.38
Intercept	−0.06	0.45	−0.93	0.81				
OBE frequency (EDE) baseline	−0.51	0.48	−1.46	0.43	0.04	0.11	< 0.01	0.26
Passive‐viewing oxygenation (fNIRS)^a^	1.10	0.48	0.17	2.04	0.12	0.11	< 0.01	0.34
Regulation oxygenation (fNIRS)^a^	1.13	0.54	0.08	2.18	0.11	0.11	< 0.01	0.33
*Rapid response models (n = 28)*								
OBE frequency (EDE) posttreatment					0.30	0.11	0.10	0.50
Intercept	1.67	0.13	1.42	1.92				
OBE frequency (EDE) baseline	0.58	0.13	0.33	0.84	0.27	0.12	0.06	0.48
Rapid response	−0.38	0.14	−0.64	−0.10	< 0.01	0.17	< 0.01	0.33
OBE frequency (EDE) follow‐up					0.17	0.14	< 0.01	0.45
Intercept	1.64	0.26	1.13	2.16				
OBE frequency (EDE) baseline	0.39	0.27	−0.14	0.92	0.14	0.18	< 0.01	0.50
Rapid response	−0.23	0.33	−0.88	0.42	0.02	0.26	< 0.01	0.53
Abstinence from binge eating (EDE) posttreatment					0.19	0.10	0.02	0.39
Intercept	−2.32	0.52	−3.38	−1.36				
OBE frequency (EDE) baseline	0.01	0.34	−0.68	0.67	0.01	0.15	< 0.01	0.30
Rapid response	1.13	0.40	0.34	1.92	0.16	0.11	< 0.01	0.37
Abstinence from binge eating (EDE) follow‐up					0.11	0.05	0.01	0.22
Intercept	−0.30	0.29	−0.87	0.25				
OBE frequency (EDE) baseline	−0.44	0.31	−1.06	0.14	0.04	0.08	< 0.01	0.18
Rapid response^a^	0.66	0.31	0.05	1.28	0.09	0.06	< 0.01	0.20

Abbreviations: CrI = credible interval; deoxygenation = percentage of trials with decreasing oxyhemoglobin levels (*β* < −0.2); EDE = eating disorder examination (Hilbert and Tuschen‐Caffier 2016a); EEG = electroencephalography; fNIRS = functional near‐infrared spectroscopy; high beta = 23–28 Hz; OBE = objective binge‐eating episode; oxygenation = percentage of trials with increasing oxyhemoglobin levels (*β* > 0.2); rapid response = at least 91.67% reduction in OBE frequency at week 4 of treatment; *β* = regression parameter in the applied general linear models representing the change in oxyhemoglobin levels.

^a^ 95% CrI did include 0 in the sensitivity analysis with doubled prior standard deviations.

^b^ 95% CrI did no longer include 0 in the sensitivity analysis with halved prior standard deviations.

#### 4.2.1 Predictors of Objective Binge‐Eating Frequency

Regarding pretreatment EEG activity, high beta power during passive‐viewing trials did not predict OBE frequency at posttreatment. However, higher high beta power during passive‐viewing trials and lower high beta power during regulation trials predicted lower OBE frequency at follow‐up, demonstrating large and medium effects, respectively. Regarding pretreatment fNIRS activity, the percentage of deoxygenation during passive‐viewing trials did not predict OBE frequency at posttreatment and the percentage of oxygenation during passive‐viewing trials did not predict OBE frequency at follow‐up. RR predicted lower OBE frequency at posttreatment, demonstrating a less than small effect, but did not predict OBE frequency at follow‐up.

#### 4.2.2 Predictors of Abstinence From Binge Eating

Regarding pretreatment EEG activity, higher high beta power during passive‐viewing trials predicted greater abstinence from binge eating at posttreatment, demonstrating a medium effect. Higher high beta power during passive‐viewing trials and lower high beta power during transfer trials predicted greater abstinence at follow‐up, demonstrating large and small effects, respectively. Regarding pretreatment fNIRS activity, a higher percentage of deoxygenation during transfer trials predicted greater abstinence at posttreatment, demonstrating a medium effect, while the percentage of oxygenation during passive‐viewing trials did not. Neither the percentage of oxygenation during passive‐viewing trials nor the percentage of oxygenation during regulation trials predicted abstinence at follow‐up. RR predicted greater abstinence at posttreatment, demonstrating a medium effect, but did not predict abstinence at follow‐up.

#### 4.2.3 Predictors of Secondary Outcomes

To summarize, regarding pretreatment EEG activity, lower high beta power during regulation trials predicted lower BMI at posttreatment and lower food cravings at both posttreatment and follow‐up, while higher high beta power during transfer trials predicted lower food cravings at posttreatment and follow‐up. Regarding pretreatment fNIRS activity, a higher percentage of deoxygenation during passive‐viewing trials predicted higher food cravings at posttreatment and higher mental quality of life at follow‐up, while a higher percentage of oxygenation during transfer trials predicted lower anxiety symptoms at posttreatment and lower waist‐to‐hip ratio at posttreatment. RR predicted lower food cravings at posttreatment.

## 5 Discussion

This preregistered study uniquely explored pretreatment neurophysiological EEG and fNIRS activity as predictors of outcomes of food‐specific high‐beta EEG‐NF or individualized food‐specific rtfNIRS‐NF in *N* = 47 individuals with BED at posttreatment and 6‐month follow‐up. The findings suggest that outcomes of NF for BED, a novel treatment approach possibly addressing limitations of current BED treatments (Bartholdy et al. 2013), can be predicted before treatment, which represents a concrete step toward personalized therapy concepts. Regarding pretreatment EEG activity, higher fronto‐central high beta power (23–28 Hz) in response to passive‐viewing of food pictures and lower high beta power during the two regulatory tasks predicted lower OBE frequency at follow‐up and greater abstinence from binge eating at posttreatment and follow‐up, suggesting greater improvements for individuals showing increased attention in response to food stimuli but also successful attention regulation. Regarding pretreatment fNIRS activity, a higher percentage of deoxygenation during the regulatory transfer trials predicted greater abstinence from binge eating at posttreatment, suggesting greater improvements for individuals showing difficulties in prefrontal regulation. Overall, neurophysiological predictors showed larger prognostic effects than rapid response (RR), the currently most consistent predictor of psychological and medical BED treatment outcomes (Vall and Wade 2015), highlighting their potential to guide future treatment allocation.

### 5.1 Pretreatment EEG Activity

In the EEG‐NF, higher pretreatment high beta power during passive‐viewing trials predicted greater abstinence from binge eating at both posttreatment and follow‐up, as well as lower OBE frequency at follow‐up, suggesting that individuals exhibiting increased attention in response to food stimuli benefited more from EEG‐NF, likely due to greater room for improvement in terms of downregulation. Notably, individuals with BED generally exhibit higher high beta power in response to food stimuli than healthy controls (Blume et al. 2019). Thus, the findings partly align with Gevensleben et al. (2009), who reported children with higher pretreatment theta power—also reflecting greater deviations from healthy norms—showing greater reductions in attention‐deficit/hyperactivity symptoms after theta downregulation and beta upregulation NF. In contrast, Kopřivová et al. (2013) reported adults with lower pretreatment delta power—reflecting smaller deviations from healthy norms—showing greater reductions in obsessive‐compulsive symptoms following delta‐theta or low‐beta downregulation NF. This discrepancy may reflect that, although Kopřivová et al. (2013) used individualized—partly non‐delta—frequency bands for NF training (e.g., 13–15 Hz), broad, non‐individualized delta activity (1–6 Hz) served as the predictor. However, although their protocol did not target high beta, they also found that higher pretreatment high beta power predicted better outcomes, which could suggest elevated high beta power as a general predictor of better NF outcomes. Nevertheless, it should be noted that both Gevensleben et al. (2009) and Kopřivová et al. (2013)—as opposed to the present study—used resting‐state EEG activity as predictors and that research regarding EEG activity downregulation paradigms remains limited (Weber et al. 2020), precluding any definite conclusions.

Lower pretreatment high beta power during regulation trials predicted lower OBE frequency at follow‐up, lower BMI at posttreatment, and lower food cravings at both posttreatment and follow‐up, suggesting that participants initially successful in utilizing visual feedback to reduce food‐related attention benefited more from EEG‐NF. Similarly, lower pretreatment high beta power during transfer trials predicted greater abstinence from binge eating at follow‐up, suggesting participants quickly reducing food‐related attention even without visual feedback were more likely to fully remit from binge eating. However, these participants also reported higher food cravings at posttreatment and follow‐up, which could reflect that abstaining from excessive food consumption—particularly from hyperpalatable foods commonly consumed during OBEs—often increases cravings (Bjorlie et al. 2022; Gordon et al. 2018; Pretlow 2011). Additionally, lower high beta power during both regulation and transfer trials showed up to large‐sized correlations with baseline characteristics known to predict favorable outcomes in eating disorder treatments (Vall and Wade 2015), including lower depressive symptoms and greater motivation (see Table S3). This suggests that adaptive attention regulation may reflect a broader trait cluster linked to greater therapy responsiveness in BED.

Overall, individuals who initially showed strong involuntary neurophysiological responses to food cues but also succeeded in voluntarily downregulating these benefited most from EEG‐NF, implying that the beneficial effect of EEG‐NF may primarily lie in reducing the involuntarily increased attention in response to food stimuli common in BED (Blume et al. 2019), rather than enhancing voluntary attention regulation. Supporting this, supplementary analyses examining the change in passive‐viewing high beta power between the first and the last attended NF session (see Table S5) revealed an association between a greater reduction in high beta power and greater abstinence at posttreatment, whereas no such association was found for transfer high beta power. This indicates that although there was no mean reduction in passive‐viewing high beta power baseline vs. posttreatment (Hilbert et al. 2024), participants showing stronger reductions in passive‐viewing high beta power were more likely to achieve abstinence.

### 5.2 Pretreatment fNIRS Activity

In rtfNIRS‐NF, a higher percentage of pretreatment deoxygenation during transfer trials predicted greater abstinence from binge eating at posttreatment, implying that individuals who—despite attempting to recruit cognitive control—experienced a decrease in control when confronted with food pictures benefited more from rtfNIRS‐NF, likely due to greater room for improvement. This partially aligns with Ho et al. (2020), who reported greater symptom reductions in patients with major depressive disorder showing lower prefrontal oxygenation during cognitive tasks at pretreatment. Conversely, improvements were also observed in participants showing a higher percentage of oxygenation in the transfer task, which predicted lower anxiety symptoms and lower waist‐to‐hip ratio at posttreatment. These participants—in contrast to those showing more deoxygenation—also had a shorter illness duration, lower OBE frequency, fewer cravings, lower BMI, and lower waist‐to‐hip ratio at baseline (see Table S4), suggesting a less severe, earlier stage of the disorder. As they seemingly already (or still) possessed effective self‐regulation strategies, their reduction in anxiety may reflect increased perceived controllability or self‐validation following early successes in the NF tasks (Bartholdy et al. 2013; Kollmann et al. 2024). This may also account for their reduced waist‐to‐hip ratio, which would be difficult to achieve within eight weeks for individuals only beginning to develop self‐regulation skills during the intervention.

A higher percentage of deoxygenation during passive‐viewing trials at pretreatment predicted higher food cravings at posttreatment but also higher mental quality of life at follow‐up, implying a delayed treatment response in participants who exhibited more involuntary decreases in cognitive control when confronted with food pictures. Although the present study used pictures of food, these findings may align with a meta‐analysis demonstrating that in vivo exposure to food transiently increases food cravings, especially among individuals with high baseline cravings, but reduces them in the long term (Wolz et al. 2020). This is further supported by medium‐sized correlations showing that individuals with a higher percentage of pretreatment deoxygenation during passive‐viewing trials showed higher baseline food cravings (see Table S4).

Overall, individuals exhibiting more pretreatment difficulties in the voluntary recruitment of food‐related cognitive control showed greater improvements in primary outcomes following rtfNIRS‐NF, whereas involuntary prefrontal responses at pretreatment did not predict primary outcomes. This suggests that the beneficial effects of rtfNIRS‐NF may primarily result from enhancing voluntary recruitment of cognitive control rather than reducing the involuntary control deficits common in BED (Rösch et al. 2021). Supporting this, supplementary analyses examining the change in transfer deoxygenation between first and the last attended NF session (see Table S5) revealed a small‐sized association between a greater reduction in deoxygenation and greater abstinence at posttreatment, whereas no such association was found for reductions in the passive‐viewing trials. While Rösch et al. (Rösch, Schmidt, Wimmer, et al. 2023) did find a nonsignificant small‐sized association between less session‐wise improvements in offline transfer regulation success and greater reductions in binge eating in the same sample, they used a different definition of regulation success—calculated as the difference in oxygenation between passive‐viewing and transfer trials—mixing involuntary and voluntary prefrontal responses.

## 6 Strengths and Limitations

The study's strengths include its novel use of neurophysiological activity as a predictor for eating disorder therapy outcomes, alongside the consideration of various outcome measures covering eating disorder and general psychopathology symptoms, all assessed with clinical interviews or validated questionnaires conducted by blind assessors. Moreover, standardized treatment protocols were implemented for both rtfNIRS‐ and EEG‐NF, treatments relatively understudied in eating disorders. Analyses were preregistered and conducted using Bayesian statistics, offering significant advantages over traditional frequentist null‐hypothesis testing (Wagenmakers et al. 2018). However, the relatively small sample consisted predominantly of white, middle‐aged women with BED, limiting generalizability to other demographics. In addition to the small sample size, the lack of available external information for the Bayesian priors—due to scarce research in this field—may have contributed to the disappearance of effects in the sensitivity analyses. Accordingly, results remain preliminary.

## 7 Conclusions

While pretreatment EEG activity showed the largest predictive effects, neurophysiological predictors overall demonstrated larger effects than RR and baseline values of therapy outcomes (Rösch, Schmidt, and Hilbert 2023). This aligns with meta‐analytic findings that parameters targeted during NF are the most reliable pretreatment predictors of NF outcomes (Weber et al. 2020). Further, the findings suggest that EEG‐NF particularly benefited individuals who exhibited increased attention in response to food stimuli but also demonstrated effective attention regulation, as indicated by higher high beta power during food‐cue exposure and lower high beta power during the regulatory tasks, respectively. In contrast, rtfNIRS‐NF particularly benefited participants exhibiting pretreatment difficulties in prefrontal regulation, as indicated by more deoxygenation during the regulatory transfer task. This difference may be explained by EEG‐NF primarily addressing involuntary attentional shifts, whereas rtfNIRS‐NF may primarily address deficits in the voluntary recruitment of cognitive control.

Research on NF is still emerging, and further development is needed before definitive clinical recommendations can be made. However, in settings with limited capacities, facilities implementing EEG‐ or rtfNIRS‐NF for BED may use these predictors to prioritize patients most likely to benefit. Future research should validate the findings in larger, more diverse samples, compare simultaneous EEG and fNIRS measurements to assess cross‐predictiveness across NF protocols, explore the neurophysiological mechanisms of action for symptom reductions, evaluate the predictive value of neurophysiological activity in non‐NF treatments for BED, further refine NF protocols, and investigate potential benefits of integrating NF with psychotherapy.

## Author Contributions

**Ben Schreglmann:** conceptualization, methodology, data curation, formal analysis, writing – original draft, writing – review and editing. **Ricarda Schmidt:** investigation, methodology, project administration, supervision, writing – review and editing. **Michael Lührs:** methodology, software, writing – review and editing. **Anja Hilbert:** conceptualization, funding acquisition, methodology, project administration, resources, supervision, writing – review and editing.

## Funding

Data collection was supported by the German Federal Ministry of Education and Research (BMBF; grant 01EO1501).

## Conflicts of Interest

Anja Hilbert reports receiving research grants from the German Federal Ministry of Education and Research, German Research Foundation, Innovation Fund, and Roland Ernst Foundation for Health Care; royalties for books on the treatment of eating disorders and obesity with Hogrefe and Kohlhammer; honoraria for workshops and lectures on eating disorders and obesity and their treatment, including for Lilly, Novo Nordisk, and Rhythm; honoraria as editor of the *International Journal of Eating Disorders*; and honoraria as a consultant for Takeda and Medice. Michael Lührs is employed by Brain Innovation B.V. at the time of writing this manuscript. The authors acknowledge no other conflicts of interest.

## Supporting information


**Data S1:** Supporting Information.

## Data Availability

The essential parts of the code used for statistical analysis and questionnaire score calculations are included in the Supporting Information or were previously published in an open‐access paper (Hilbert et al. 2024). Additional code can be made available upon request to the corresponding author. Datasets are not publicly available due to the sensitive nature of the patient information included but will be made available upon reasonable request to Professor Anja Hilbert.
