# Supplementary material for: Neurofeedback for Binge‐Eating Disorder: Neurophysiological Outcome Predictors and Rapid Response
Source: Int J Eat Disord. 2026 Jan 7;59(4):766–77. doi: 10.1002/eat.70023 (PMC13058408; doi:10.1002/eat.70023)
Supplement: Supplementary file 1 — Data S1: Supporting Information. [file EAT-59-766-s001.docx]

| **Supplementary Material**  Neurofeedback for binge-eating disorder:  Neurophysiological outcome predictors and rapid response  **NIRSBED** |
| --- |

Authors: Ben Schreglmann^1^; Ricarda Schmidt^1^; Michael Lührs^2,3^; Anja Hilbert^1^

^1^Integrated Research and Treatment Center AdiposityDiseases, Behavioral Medicine Research Unit, Department of Psychosomatic Medicine and Psychotherapy, Leipzig University Medical Center, Leipzig, Germany

^2^Faculty of Psychology and Neuroscience, Maastricht University, Maastricht, The Netherlands

^3^Brain Innovation B.V., Maastricht, The Netherlands

**Supplementary Method**

**EEG data preprocessing**

For data recording the NEURO PRAX® EEG—full-band DC-EEG Bio- and Neurofeedback-System by Neurocare (THERA PRAX® neuroConn GmbH, Ilmenau, Germany) was used. The data were recorded from the Fz, Cz, Fc1, and Fc2 electrodes, referenced to the mastoids as per the international 10-20 system (Chatrian et al., 1985), at a sampling rate of 256 Hz. EEG data were preprocessed using a band-pass filter and a notch filter. The continuous signal was segmented into 2-second intervals, and ocular artifacts were corrected. Automatic artifact rejection removed segments with voltage steps exceeding 50µV/ms or amplitudes outside ±100µV. Subsequently, a highly trained researcher visually inspected the data to remove remaining artifacts, such as focal abnormalities or signs of drowsiness. Recordings from the passive-viewing trials with <25 artifact-free segments were excluded. The filtered data were then subjected to Fourier transformation with a 20% Hanning window, extracting the high beta (23–28Hz) frequency band.

**fNIRS data preprocessing**

Data were recorded using the NIRScout, a 28-channel continuous-wave NIRS system provided by NIRStar Software version 15.0 (NIRx Medizintechnik GmbH, Berlin, Germany) with a 7.8125 Hz sampling rate, while online data preprocessing and NF calculation were performed using Turbo-Satori version 1.0.0 (Lührs & Goebel, 2017). The 8 light sources and 12 light detectors were spread over the prefrontal cortex in 3.0cm distances, except two that were 4.5 and 5.5cm. The Fz electrode served as reference, and detectors 1 and 2 aligned with the nasion-inion line as per the international 10-20 system (Chatrian et al., 1985). In Satori version 2.0.6 (Brain Innovation B.V. & NIRx Medical Technologies, 2024) data was converted from raw wavelength (760;850nM) signals to oxyhemoglobin levels utilizing the modified Beer-Lambert law (Delpy et al., 1988), then z-transformed and filtered with a high-pass filter at 0.005 Hz and a low-pass filter at 0.4 Hz. The coefficient of variation (CV) for each recording was calculated using CV(%)=100×standard deviation/mean. Recordings with a CV>7.5% in one or both training channels were excluded.

**Rapid response and predictors of rapid response**

To define rapid response (RR), OBE frequency over the past seven days was assessed at the beginning of each training session with a modified EDE‑Q item (see Supplementary Table S1). Following the work of Grilo et al. (2006) and Grilo and Masheb (2007), the cutoff value of proportional reduction in OBE frequency at weeks 1, 2, 3 or 4 of treatment that best predicted abstinence from binge eating at posttreatment was evaluated by employing four receiver operating characteristic (ROC) analyses (Robin et al., 2011) and data from both NF groups. The proportional reduction at the respective timepoint was calculated using the formula:


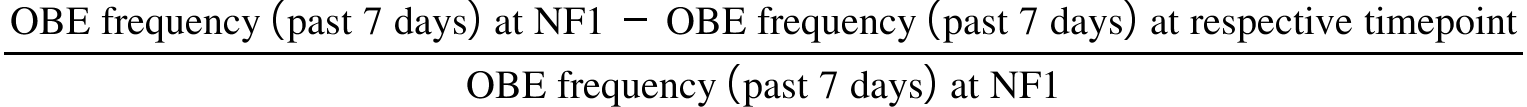


For each of the four ROC analyses, only data from participants whose OBE frequency was assessed at least once in the respective timeframe (day 7, 14, 21, or 28 of treatment ± 3 days) were used, with first training session being day 0. If there was more than one assessed value in the given timeframe, the more recent value closest to the respective week mark (7, 14, 21, or 28) was used. The ROC curve for week 4 yielded the greatest area under the curve (see Supplementary Table S9) with a large effect according to Rice and Harris (2005; small effect, ≥.556; moderate, ≥.638; large, ≥.714) at a reduction in OBE frequency of 91.67%. Of the *n*=28 participants (EEG-NF *n*=14, rtfNIRS-NF *n*=14) that were assessed at least once within the timeframe used for defining RR (days 25**–**31 of treatment), *n*=8 (EEG-NF *n*=2, rtfNIRS-NF *n*=6) or 28.57% (EEG-NF 14.29%, rtfNIRS-NF 42.86%) showed a RR.

Further, a Bayesian logistic regression was employed to assess baseline and pretreatment predictors of RR, including EEG predictors, fNIRS predictors, sociodemographics, and baseline values of primary and secondary outcomes, as well as several additional baseline variables that were assessed via patient-report at baseline, including motivation to change eating behavior (10-point Likert scale ranging from 1=“not at all” to 10=“completely”), motivation to maintain change long term (10-point Likert scale ranging from 1=“not at all” to 10=“completely”), confidence to maintain change long term (10-point Likert scale ranging from 1=“not at all” to 10=“completely”), impulsivity (Behavioral Inhibition System/Behavioral Activation System Questionnaire [BIS/BAS]; BIS: 1**–**4, BAS: 1**–**4; Strobel et al., 2006), difficulties in emotion regulation (Difficulties in Emotion Regulation Scale [DERS]; Gutzweiler & In-Albon, 2018), self-efficacy (Generalized Self-Efficacy Scale [GSES]; Jerusalem & Schwarzer, 2003), sleep quality (Pittsburgh Sleep Quality Index [PSQI]; Backhaus et al., 2002), and perceived stress (Perceived Stress Scale [PSS-10]; Klein et al., 2016). Higher scores indicated greater impairment, except for the GSES (higher scores=lower impairment), therapy expectations (higher scores=more optimistic expectations), BIS (higher scores=greater inhibition), and BAS (higher scores=greater activation). All questionnaires were administered in German versions. Baseline values can be found in Supplementary Table S10. Internal consistencies for the DERS (α=.95, ω=.96) and GSES (α=.92, ω=.92), as well as the code for calculating the DERS, GSES, and BIS/BAS scores, were reported by Hilbert et al. (2024) in their Supplementary Material. Internal consistencies for the BIS (α=.81, ω=.81) and BAS (α=.71, ω=.72) subscales, the PSQI (α=.76, ω=.78), and the PSS-10 (α=.89, ω=.90) were calculated in the present study. The corresponding code for PSQI and PSS-10 score computation is provided below. NF group (EEG-NF, rtfNIRS-NF) was included as moderator.

**R code**

**Calculate Bayesian linear models using imputed datasets**

# Empirical-Bayes runner across multiple imputations

#

# Overview:

# - For each imputed dataset, fit a frequentist baseline model to obtain MLEs and SEs.

# - Construct empirical-Bayes priors from those MLEs/SEs and (optionally) fit the Bayesian

# analogue with brms. Numeric predictors on the RHS are z-standardized within each imputation.

# - Compute PSIS-LOO via loo::loo() with moment_match = TRUE and reloo = TRUE for Bayesian fits only.

#

# Supported model_type:

# "logReg" : logistic regression (Bernoulli, logit)

# "linReg" : linear regression (Gaussian)

# "GLMpoi" : Poisson regression (log link)

# "GLMnegbin" : Negative Binomial regression (log link)

# "GLMbeta" : Beta regression (logit link for the mean)

#

# Inputs:

# - formula_str : character, e.g., "y ~ x1 + x2"

# - imputed_data : list of data.frames, one per imputation

# - model_type : one of the strings above

# - do_bayes : logical; if TRUE, fit Bayesian model(s) with EB priors

# - iter, warmup : MCMC iterations per chain and warmup

# - max_treedepth : Stan HMC maximum tree depth

# - adapt_delta : Stan target acceptance probability

# - chains, thin : number of chains; thinning factor

# - init : "empirical" to initialize from frequentist MLEs; or pass a brms init

# - prior_multiplier : optional scalar to widen/narrow prior SDs (e.g., for sensitivity analysis)

#

# Priors used (Bayesian fits):

# - Coefficients: Normal(mean = MLE, sd = SE); fallback Normal(0, default_sd)

# - linReg sigma: Student-t(3, location = MLE sigma, scale ≈ sigma / sqrt(2*df))

# - GLMnegbin shape (theta): Normal on log(theta) with sd ≈ SE.theta / theta

# - GLMbeta precision (phi): Normal on log(phi) intercept (robustly extracted)

#

# Outputs (returned list):

# - fitfreq : list of frequentist fits per imputation

# - fitbayes : list of brmsfit objects per imputation (NULLs if do_bayes = FALSE)

# - GVIFs : per-imputation (G)VIF tables (for betareg: NA)

# - GVIF_means : averaged (G)VIF across imputations

# - ELPD_LOO : numeric vector of elpd-LOO per imputation (Bayesian only)

# - ELPD_LOO_mean : mean(ELPD, na.rm = TRUE)

# - LOOIC : numeric vector of LOOIC per imputation (= -2 * ELPD)

# - LOOIC_mean : mean(LOOIC, na.rm = TRUE)

# - freq_pool : Rubin-pooled frequentist summary (coef, vcov, W, B)

# - bayes_draws : posterior draws stacked across imputations; includes column .imp

#

# Notes:

# - LOO metrics are computed only if do_bayes = TRUE; otherwise ELPD/LOOIC are NA.

# - For GLMbeta, car::vif is unreliable; GVIF entry is set to NA.

# - Default prior SD for fallback normals is 10 * prior_multiplier (if provided).

run_models <- function(formula_str, imputed_data, model_type, do_bayes,

iter, warmup, max_treedepth, adapt_delta,

chains, thin, init, prior_multiplier = NULL) {

# ---- Packages -------------------------------------------------------------

suppressPackageStartupMessages({

library(car)

if (do_bayes) { library(brms); library(loo); library(posterior) }

})

`%||%` <- function(x, y) if (is.null(x)) y else x

cores <- max(1, parallel::detectCores())

safe_vif <- function(model) tryCatch(car::vif(model), error = function(e) NA)

extract_coef_vcov <- function(model, type) {

if (type %in% c("linReg", "logReg", "GLMpoi", "GLMnegbin")) {

list(coef = coef(model), vcov = vcov(model))

} else if (type == "GLMbeta") {

list(coef = coef(model, model = "mean"),

vcov = vcov(model, model = "mean"))

} else stop("Unknown model_type in extractor.")

}

rubin_pool <- function(models, type) {

m <- length(models)

ex <- lapply(models, extract_coef_vcov, type = type)

common <- Reduce(intersect, lapply(ex, \(e) names(e$coef)))

if (!length(common)) return(NULL)

Bmat <- do.call(cbind, lapply(ex, \(e) e$coef[common]))

Uls <- lapply(ex, \(e) e$vcov[common, common, drop = FALSE])

bbar <- rowMeans(Bmat, na.rm = TRUE)

W <- Reduce(`+`, Uls) / m

B <- stats::cov(t(Bmat))

Tcov <- W + (1 + 1/m) * B

list(coef = bbar, vcov = Tcov, W = W, B = B)

}

build_normal_priors <- function(coef_mle, se_mle, default_sd) {

pri <- set_prior(paste0("normal(0, ", default_sd, ")"), class = "Intercept")

if (!is.na(coef_mle["(Intercept)"]) && !is.na(se_mle["(Intercept)"])) {

pri <- set_prior(

paste0("normal(", coef_mle["(Intercept)"], ", ", se_mle["(Intercept)"], ")"),

class = "Intercept"

)

}

for (nm in names(coef_mle)[-1]) {

if (!is.na(coef_mle[nm]) && !is.na(se_mle[nm])) {

pri <- c(pri, set_prior(paste0("normal(", coef_mle[nm], ", ", se_mle[nm], ")"),

class = "b", coef = nm))

} else {

pri <- c(pri, set_prior(paste0("normal(0, ", default_sd, ")"),

class = "b", coef = nm))

}

}

pri

}

stack_draws <- function(fits) {

if (!length(fits)) return(NULL)

dl <- lapply(seq_along(fits), function(i) {

fi <- fits[[i]]; if (is.null(fi)) return(NULL)

dd <- posterior::as_draws_df(fi); dd$.imp <- i; dd

})

dl <- Filter(Negate(is.null), dl)

if (!length(dl)) NULL else do.call(rbind, dl)

}

make_vif_table <- function(model, n_pred) {

if (n_pred < 2) return(NA)

v <- safe_vif(model)

if (is.data.frame(v) || is.matrix(v)) {

gv <- v[, "GVIF"]; df <- v[, "Df"]

data.frame(Term = rownames(v), GVIF = gv, Df = df,

Adjusted_GVIF = gv^(1 / (2 * df)))

} else if (is.atomic(v)) {

data.frame(Term = names(v), VIF = as.numeric(v))

} else NA

}

# ---- Setup ----------------------------------------------------------------

fmla <- as.formula(formula_str)

n_imp <- length(imputed_data)

freq_fits <- vector("list", n_imp)

bayes_fits <- vector("list", n_imp)

vif_tables <- vector("list", n_imp)

# LOO containers (Bayesian only)

looic_vec <- rep(NA_real_, n_imp) # -2 * elpd_loo

elpd_vec <- rep(NA_real_, n_imp) # elpd_loo

# ---- Loop over imputations ------------------------------------------------

for (i in seq_len(n_imp)) {

df <- imputed_data[[i]]

# z-standardize numeric RHS predictors within each imputation

rhs_vars <- all.vars(fmla[[3]])

for (v in rhs_vars) if (is.numeric(df[[v]])) df[[v]] <- scale(df[[v]])

default_sd <- 10 * (prior_multiplier %||% 1)

if (model_type == "logReg") {

m_freq <- glm(fmla, data = df, family = binomial())

freq_fits[[i]] <- m_freq

if (do_bayes) {

b <- coef(m_freq); se <- sqrt(diag(vcov(m_freq)))

if (!is.null(prior_multiplier)) se <- se * prior_multiplier

pri <- build_normal_priors(b, se, default_sd)

init_fun <- if (identical(init, "empirical")) {

function() list(b = as.numeric(b[-1]),

Intercept = as.numeric(b["(Intercept)"]))

} else init

bayes_fits[[i]] <- brm(

fmla, data = df, family = bernoulli(),

prior = pri, chains = chains, iter = iter, warmup = warmup,

cores = cores, seed = 42,

control = list(adapt_delta = adapt_delta,

max_treedepth = max_treedepth),

thin = thin, init = init_fun

)

}

vif_tables[[i]] <- make_vif_table(m_freq, length(rhs_vars))

} else if (model_type == "linReg") {

m_freq <- lm(fmla, data = df); freq_fits[[i]] <- m_freq

if (do_bayes) {

b <- coef(m_freq); se <- sqrt(diag(vcov(m_freq)))

if (!is.null(prior_multiplier)) se <- se * prior_multiplier

pri <- build_normal_priors(b, se, default_sd)

sig <- summary(m_freq)$sigma

df_r <- df.residual(m_freq)

sig_se <- (sig / sqrt(2 * df_r)) * (prior_multiplier %||% 1)

pri <- c(pri, set_prior(paste0("student_t(3, ", sig, ", ", sig_se, ")"),

class = "sigma"))

init_fun <- if (identical(init, "empirical")) {

function() list(b = as.numeric(b[-1]),

Intercept = as.numeric(b["(Intercept)"]),

sigma = sig)

} else init

bayes_fits[[i]] <- brm(

fmla, data = df, family = gaussian(),

prior = pri, chains = chains, iter = iter, warmup = warmup,

cores = cores, seed = 42,

control = list(adapt_delta = adapt_delta,

max_treedepth = max_treedepth),

thin = thin, init = init_fun

)

}

vif_tables[[i]] <- make_vif_table(m_freq, length(rhs_vars))

} else if (model_type == "GLMpoi") {

m_freq <- glm(fmla, data = df, family = poisson(link = "log"))

freq_fits[[i]] <- m_freq

if (do_bayes) {

b <- coef(m_freq); se <- sqrt(diag(vcov(m_freq)))

if (!is.null(prior_multiplier)) se <- se * prior_multiplier

pri <- build_normal_priors(b, se, default_sd)

init_fun <- if (identical(init, "empirical")) {

function() list(b = as.numeric(b[-1]),

Intercept = as.numeric(b["(Intercept)"]))

} else init

bayes_fits[[i]] <- brm(

fmla, data = df, family = poisson(link = "log"),

prior = pri, chains = chains, iter = iter, warmup = warmup,

cores = cores, seed = 42,

control = list(adapt_delta = adapt_delta,

max_treedepth = max_treedepth),

thin = thin, init = init_fun

)

}

vif_tables[[i]] <- make_vif_table(m_freq, length(rhs_vars))

} else if (model_type == "GLMnegbin") {

suppressPackageStartupMessages(library(MASS))

m_freq <- MASS::glm.nb(fmla, data = df, link = log)

freq_fits[[i]] <- m_freq

if (do_bayes) {

b <- coef(m_freq); se <- sqrt(diag(vcov(m_freq)))

if (!is.null(prior_multiplier)) se <- se * prior_multiplier

pri <- build_normal_priors(b, se, default_sd)

theta <- m_freq$theta

theta_se <- m_freq$SE.theta * (prior_multiplier %||% 1)

mu_eta <- log(theta); sd_eta <- theta_se / theta

pri <- c(pri, set_prior(sprintf("normal(%f,%f)", mu_eta, sd_eta),

class = "shape"))

init_fun <- if (identical(init, "empirical")) {

function() list(b = as.numeric(b[-1]),

Intercept = as.numeric(b["(Intercept)"]))

} else init

bayes_fits[[i]] <- brm(

fmla, data = df, family = negbinomial(link = "log"),

prior = pri, chains = chains, iter = iter, warmup = warmup,

cores = cores, seed = 42,

control = list(adapt_delta = adapt_delta,

max_treedepth = max_treedepth),

save_pars = save_pars(all = TRUE),

thin = thin, init = init_fun

)

}

vif_tables[[i]] <- make_vif_table(m_freq, length(rhs_vars))

} else if (model_type == "GLMbeta") {

suppressPackageStartupMessages(library(betareg))

m_freq <- betareg::betareg(fmla, data = df, link = "logit")

freq_fits[[i]] <- m_freq

if (do_bayes) {

b <- coef(m_freq, model = "mean")

se <- sqrt(diag(vcov(m_freq, model = "mean")))

if (!is.null(prior_multiplier)) se <- se * prior_multiplier

pri <- build_normal_priors(b, se, default_sd)

prec_coef <- coef(m_freq, model = "precision")

prec_vcov <- vcov(m_freq, model = "precision")

candidates <- c("(Intercept)","precision.(Intercept)","phi.(Intercept)",

"Intercept","phi","log(phi)")

idx <- if (!is.null(names(prec_coef))) {

h <- which(names(prec_coef) %in% candidates); if (length(h)) h[1] else 1L

} else 1L

int_name <- if (!is.null(names(prec_coef))) names(prec_coef)[idx] else NULL

logphi_hat <- as.numeric(prec_coef[[idx]])

if (is.matrix(prec_vcov)) {

logphi_se <- if (!is.null(rownames(prec_vcov)) && !is.null(int_name) &&

int_name %in% rownames(prec_vcov)) {

sqrt(prec_vcov[int_name, int_name, drop = TRUE])

} else sqrt(prec_vcov[idx, idx, drop = TRUE])

} else {

logphi_se <- sqrt(as.numeric(prec_vcov)[idx])

}

logphi_se <- (logphi_se %||% 0.5) * (prior_multiplier %||% 1)

if (!is.finite(logphi_se) || logphi_se <= 0) logphi_se <- 0.5

pri <- c(pri, set_prior(sprintf("normal(%f,%f)", logphi_hat, logphi_se),

class = "phi"))

init_fun <- if (identical(init, "empirical")) {

function() list(b = as.numeric(b[-1]),

Intercept = as.numeric(b["(Intercept)"]))

} else init

bayes_fits[[i]] <- brm(

fmla, data = df, family = Beta(link = "logit"),

prior = pri, chains = chains, iter = iter, warmup = warmup,

cores = cores, seed = 42,

control = list(adapt_delta = adapt_delta,

max_treedepth = max_treedepth),

thin = thin, init = init_fun

)

}

vif_tables[[i]] <- NA # car::vif not reliable for betareg

} else {

stop("Invalid model_type. Use 'logReg', 'linReg', 'GLMnegbin', 'GLMbeta', 'GLMpoi'.")

}

# --- LOO metrics (Bayesian only) -----------------------------------------

if (do_bayes && !is.null(bayes_fits[[i]])) {

lo <- loo(bayes_fits[[i]], moment_match = TRUE, reloo = TRUE)

elpd_i <- lo$estimates["elpd_loo", "Estimate"]

elpd_vec[i] <- elpd_i

looic_vec[i] <- -2 * elpd_i

}

}

# ---- Aggregate VIF/GVIF across imputations --------------------------------

all_terms <- unique(unlist(lapply(vif_tables, \(x) if (is.data.frame(x)) x$Term else NULL)))

vif_means <- if (length(all_terms)) data.frame(Term = all_terms) else data.frame()

has_adj <- any(sapply(vif_tables, \(x) is.data.frame(x) && "Adjusted_GVIF" %in% names(x)))

if (nrow(vif_means)) {

if (has_adj) {

vif_means$Adjusted_GVIF <- sapply(all_terms, function(term)

mean(sapply(vif_tables, \(x)

if (is.data.frame(x) && term %in% x$Term && "Adjusted_GVIF" %in% names(x))

x$Adjusted_GVIF[x$Term == term] else NA_real_), na.rm = TRUE))

} else {

vif_means$VIF <- sapply(all_terms, function(term)

mean(sapply(vif_tables, \(x)

if (is.data.frame(x) && term %in% x$Term && "VIF" %in% names(x))

x$VIF[x$Term == term] else NA_real_), na.rm = TRUE))

}

}

# ---- Summaries ------------------------------------------------------------

pooled_freq <- rubin_pool(freq_fits, model_type)

stacked_draws <- if (do_bayes) stack_draws(bayes_fits) else NULL

list(

fitfreq = freq_fits,

fitbayes = bayes_fits,

GVIFs = vif_tables,

GVIF_means = vif_means,

# LOO metrics (Bayesian fits only)

ELPD_LOO = elpd_vec,

ELPD_LOO_mean = mean(elpd_vec, na.rm = TRUE),

LOOIC = looic_vec,

LOOIC_mean = mean(looic_vec, na.rm = TRUE),

# Other summaries

freq_pool = pooled_freq,

bayes_draws = stacked_draws

)

}

**Manually pooling Bayesian model estimates using Rubin’s Rule**

# Purpose:

# Combine parameter estimates from a set of Bayesian models fitted to multiply

# imputed datasets. For each parameter, the function pools the posterior mean

# and variance via Rubin’s rules and reports a (1−α) credible interval using a

# normal approximation. It also summarizes MCMC diagnostics (R̂, Bulk ESS, Tail ESS)

# across imputations. Finally, it pools Bayes-R² as a scalar performance metric.

#

# Inputs:

# - model_list : list of Bayesian model fits (one per imputation).

# - alpha : tail probability for credible intervals (default 0.05 → 95% CrI).

# - digits : number of decimals for rounded output (default 2).

#

# Output:

# - Data frame with one row per parameter (plus a row for Bayes_R2) containing:

# Parameter | Estimate | SE | CI_lower | CI_upper | Rhat_mean | ESS_bulk_mean | ESS_tail_mean

#

# Notes and limitations:

# - Pooling is performed on posterior means and variances using Rubin’s rules:

# total_var = mean(within_var) + (1 + 1/m) * var(within_means)

# where m is the number of imputations.

# - Credible intervals use a normal approximation around the pooled mean

# (use quantile-based intervals if strong asymmetry is expected).

# - Diagnostic extraction is designed to work with typical rstanarm/brms summaries;

# if a parameter name differs (e.g., “b_” prefixes), a simple cleanup is applied.

# - The Bayes-R² pooling assumes bayes_R2(model) returns an estimate and its SE.

# If bayes_R2 returns draws in your setup, adapt the extraction accordingly.

pool_bayesian_models <- function(model_list, alpha = 0.05, digits = 2) {

library(rstanarm)

# Basic input check

if (length(model_list) == 0) {

stop("model_list must contain at least one model.")

}

# Number of imputations

m <- length(model_list)

# Parameter names inferred from posterior draws of the first model

parameter_names <- colnames(as.data.frame(model_list[[1]]))

# Helper: extract a diagnostic (Rhat, Bulk_ESS, Tail_ESS) robustly

extract_diag <- function(mod, param, diag_name) {

mod_summary <- tryCatch(summary(mod), error = function(e) NULL)

if (is.null(mod_summary)) return(NA)

if ("fixed" %in% names(mod_summary) && param %in% rownames(mod_summary$fixed)) {

return(mod_summary$fixed[param, diag_name])

} else if ("spec_pars" %in% names(mod_summary) && param %in% rownames(mod_summary$spec_pars)) {

return(mod_summary$spec_pars[param, diag_name])

} else {

return(NA)

}

}

# Normal-approximation multiplier for (1 - alpha) credible intervals

z_score <- qnorm(1 - alpha / 2)

# Storage for per-parameter pooled results

results_list <- vector("list", length(parameter_names))

# Pool each parameter

for (i in seq_along(parameter_names)) {

original_name <- parameter_names[i]

# Harmonize typical brms/rstanarm naming (strip leading "b_" if present)

parameter_name <- ifelse(grepl("^b_", original_name),

sub("^b_", "", original_name),

original_name)

# Posterior draws for this parameter across imputations

samples_list <- lapply(model_list, function(mod) {

as.data.frame(mod)[[original_name]]

})

# Within-imputation summaries

within_means <- sapply(samples_list, mean, na.rm = TRUE)

within_vars <- sapply(samples_list, var, na.rm = TRUE)

# Rubin’s rules

pooled_mean <- mean(within_means)

between_var <- var(within_means)

avg_within_var <- mean(within_vars)

total_var <- avg_within_var + (1 + 1/m) * between_var

pooled_se <- sqrt(total_var)

# (1 − alpha) credible interval (normal approximation)

CI_lower <- pooled_mean - z_score * pooled_se

CI_upper <- pooled_mean + z_score * pooled_se

# MCMC diagnostics pooled by simple averaging across imputations

rhat_values <- sapply(model_list, function(mod) extract_diag(mod, parameter_name, "Rhat"))

ess_bulk_values <- sapply(model_list, function(mod) extract_diag(mod, parameter_name, "Bulk_ESS"))

ess_tail_values <- sapply(model_list, function(mod) extract_diag(mod, parameter_name, "Tail_ESS"))

# Assemble row

results_list[[i]] <- data.frame(

Parameter = original_name,

Estimate = round(pooled_mean, digits),

SE = round(pooled_se, digits),

CI_lower = round(CI_lower, digits),

CI_upper = round(CI_upper, digits),

Rhat_mean = round(mean(rhat_values, na.rm = TRUE), digits),

ESS_bulk_mean = round(mean(ess_bulk_values, na.rm = TRUE), digits),

ESS_tail_mean = round(mean(ess_tail_values, na.rm = TRUE), digits),

stringsAsFactors = FALSE

)

}

# Bind parameter rows

pooled_results <- do.call(rbind, results_list)

# Pooled Bayes-R² (scalar performance summary)

# Assumes bayes_R2(model) returns c(estimate, se) for each model.

bayes_r2_values <- sapply(model_list, function(mod) bayes_R2(mod)[1])

bayes_r2_within_var <- mean(sapply(model_list, function(mod) bayes_R2(mod)[2]^2))

bayes_r2_between_var <- var(bayes_r2_values, na.rm = TRUE)

bayes_r2_total_var <- bayes_r2_within_var + (1 + 1/m) * bayes_r2_between_var

bayes_r2_se <- sqrt(bayes_r2_total_var)

bayes_r2_mean <- mean(bayes_r2_values)

bayes_r2_ci_lower <- max(0, bayes_r2_mean - z_score * bayes_r2_se)

bayes_r2_ci_upper <- min(1, bayes_r2_mean + z_score * bayes_r2_se)

bayes_r2_row <- data.frame(

Parameter = "Bayes_R2",

Estimate = round(bayes_r2_mean, digits),

SE = round(bayes_r2_se, digits),

CI_lower = round(bayes_r2_ci_lower, digits),

CI_upper = round(bayes_r2_ci_upper, digits),

Rhat_mean = NA,

ESS_bulk_mean = NA,

ESS_tail_mean = NA,

stringsAsFactors = FALSE

)

# Append Bayes-R²

pooled_results <- rbind(pooled_results, bayes_r2_row)

return(pooled_results)

}

# Example:

# pooled_df <- pool_bayesian_models(results$fitbayes, alpha = 0.05, digits = 2)

# head(pooled_df)

**Computation of the PSQI score**

#' Re-code 1–4 Likert to 0–3 (numeric input assumed)

likert0_3 <- function(x) ifelse(!is.na(x), x - 1, NA_real_)

#' "HH:MM[:SS]" → minutes after midnight

min_after_midnight <- function(t) {

if (is.na(t) || t == "") return(NA_real_)

p <- as.numeric(strsplit(t, ":", fixed = TRUE)[[1]]); if (length(p) == 2L) p <- c(p, 0)

p[1] * 60 + p[2] + p[3] / 60

}

# Adds `score.psqi` and component columns; expects numeric raw items.

score_psqi <- function(df, keep = TRUE, digits = NULL) {

library(dplyr, warn.conflicts = FALSE)

df <- df %>%

mutate(

PSQI1 = sapply(PSQI1, min_after_midnight),

PSQI3 = sapply(PSQI3, min_after_midnight),

across(matches("^PSQI5[A-J]$"), likert0_3),

across(PSQI6:PSQI10, likert0_3),

PSQI5J = tidyr::replace_na(PSQI5J, 0)

) %>%

# ---- components --------------------------------------------------------

mutate(

Comp1 = PSQI6,

# sleep latency component: first derive category (0–3), add PSQI5A (0–3),

# then re‑bin raw 0–6 into 0–3 as per manual

LatCat = case_when(

PSQI2 <= 15 ~ 0,

PSQI2 <= 30 ~ 1,

PSQI2 <= 60 ~ 2,

PSQI2 > 60 ~ 3,

TRUE ~ NA_real_

),

LatRaw = LatCat + PSQI5A,

Comp2 = case_when(

LatRaw == 0 ~ 0,

LatRaw %in% 1:2 ~ 1,

LatRaw %in% 3:4 ~ 2,

LatRaw %in% 5:6 ~ 3,

TRUE ~ NA_real_

),

Comp3 = case_when(

PSQI4 >= 7 ~ 0,

PSQI4 >= 6 ~ 1,

PSQI4 >= 5 ~ 2,

PSQI4 < 5 ~ 3,

TRUE ~ NA_real_

),

time_bed = if_else(PSQI1 > PSQI3, 1440 - PSQI1 + PSQI3, PSQI3 - PSQI1),

Comp4 = case_when(

100 * PSQI4 / (time_bed / 60) >= 85 ~ 0,

100 * PSQI4 / (time_bed / 60) >= 75 ~ 1,

100 * PSQI4 / (time_bed / 60) >= 65 ~ 2,

100 * PSQI4 / (time_bed / 60) < 65 ~ 3,

TRUE ~ NA_real_

),

Comp5 = cut(rowSums(across(PSQI5B:PSQI5J, tidyr::replace_na, 0)),

c(-Inf, 0, 9, 18, Inf), labels = 0:3, right = TRUE) |> as.numeric(),

Comp6 = PSQI7,

Comp7 = cut(PSQI8 + PSQI9, c(-Inf, 0, 2, 4, Inf), labels = 0:3, right = TRUE) |> as.numeric(),

score.psqi = Comp1 + Comp2 + Comp3 + Comp4 + Comp5 + Comp6 + Comp7

) %>%

mutate(score.psqi = if_else(rowSums(is.na(select(., starts_with("Comp")))) > 0,

NA_real_, score.psqi))

if (!keep) df <- df %>% select(-matches("^PSQI|^Comp|time_bed|LatCat|LatRaw"))

if (!is.null(digits)) df <- df %>% mutate(score.psqi = round(score.psqi, digits))

df

}

**Computation of the PSS-10 score**

score_pss10 <- function(df, keep = TRUE, digits = NULL) {

library(dplyr, warn.conflicts = FALSE)

items <- paste0("PSS", 1:10)

rev <- paste0("PSS", c(4, 5, 7, 8))

df <- df %>%

mutate(across(all_of(rev), ~ 6 - .x), # proper reverse‑coding

nvalid.pss = rowSums(!is.na(across(all_of(items)))),

score.pss = if_else(nvalid.pss <= 2,

(rowMeans(across(all_of(items)), na.rm = TRUE) * 10) - 10,

NA_real_))

if (!keep) df <- df %>% select(-all_of(items), -nvalid.pss)

if (!is.null(digits)) df <- df %>% mutate(score.pss = round(score.pss, digits))

df

}

**Supplementary Tables**

**Table S1**

*Overview of measures*

| Construct | Measure | Computation | Assessment | Valid values (*n*) | Imputed values (*n*) |
| --- | --- | --- | --- | --- | --- |
| *Eating-disorder related variables* | | | |  |  |
| Illness duration | Interview | Months | t0 | 43 | 0 |
| Abstinence from binge eating | EDE | 0 OBEs, >0 OBEs over the past 28 days | t0  t1  t2 | 47  45  42 | 0  2  5 |
| OBE frequency (past 28 days) | EDE | Cumulative number of objective binge-eating episodes over the past 28 days | t0  t1  t2 | 47  45  42 | 0  2  5 |
| Eating disorder psychopathology | EDE-Q | Mean global score (0–6) | t0  t1  t2 | 45  44  41 | 2  3  6 |
| OBE frequency (past 7 days) | Modified EDE-Q item | “Over the past 7 days, how many times have you eaten what other people would regards as an unusually large amount of food (given the circumstances)? … On how many of these times did you have a sense of having lost control over your eating (at the time you were eating)?” | Before each training session at week marks 1–4 | w1: 32  w2: 30  w3: 31  w4: 28 | -  -  -  - |
| Rapid response | - | Reduction of ≥91.67% in OBE frequency (past 7 days) at week 4, defined via ROC analyses predicting abstinence from binge eating at post treatment | - | - | - |
| Food cravings | FCQ-T-r | Sum score (15–75) | t0  t1  t2 | 44  44  41 | 3  3  6 |
| Body mass index | - | Calculated from objectively measured height and weight (kg/m^2^) | t0  t1  t2 | 47  45  41 | 0  2  6 |
| Waist-to-hip ratio | - | Calculated from objectively measured waist and hip circumference | t0  t1  t2 | 46  36  32 | 1  11  15 |
| *Further psychopathology related variables* | | | |  |  |
| Depressive symptoms | PHQ-9 | Sum score (0–27) | t0  t1  t2 | 45  44  41 | 2  3  6 |
| Anxiety symptoms | GAD-7 | Sum score (0–21) | t0  t1  t2 | 45  44  41 | 2  3  6 |
| Quality of life | SF-12 | Standardized, weighted sum scores for the Physical Component Summary and the Mental Component Summary | t0  t1  t2 | 45  43  40 | 2  4  7 |
| Impulsivity | BIS/BAS | Mean scores for the BIS and the BAS (1–4) | t0 | 45 | 2 |
| Difficulties in emotion regulation | DERS | Global sum score (36–180) | t0 | 45 | 2 |
| Self-efficacy | GSES | Sum score (10–40) | t0 | 45 | 2 |
| Sleep quality | PSQI | Global sum score (0–21) | t0 | 43 | 4 |
| Perceived stress | PSS-10 | Global sum score (0–40) | t0 | 45 | 2 |
| *Neurophysiological variables* | | | |  |  |
| EEG activity |  |  | pretreatment |  |  |
| Passive-viewing trials | EEG | Mean fronto-central high beta power  during 90s of passive-viewing food-cue presentation (µV²) |  | 22 | 0 |
| Regulation trials | EEG | Mean fronto-central high beta power  during regulation trials (µV²) |  | 21 | 0 |
| Transfer trials | EEG | Mean fronto-central high beta power  during transfer trials (µV²) |  | 21 | 0 |
| fNIRS activity |  |  | pretreatment |  |  |
| Passive-viewing trials |  |  |  |  |  |
| Oxygenation | fNIRS | Percentage of mirror trials with GLM *β*>0.2 |  | 22 | 0 |
| Deoxygenation | fNIRS | Percentage of mirror trials with GLM *β*<-0.2 |  | 22 | 0 |
| Regulation trials |  |  |  |  |  |
| Oxygenation | fNIRS | Percentage of regulation trials with GLM *β*>0.2 |  | 22 | 0 |
| Deoxygenation | fNIRS | Percentage of regulation trials with GLM *β*<-0.2 |  | 22 | 0 |
| Transfer trials |  |  |  |  |  |
| Oxygenation | fNIRS | Percentage of transfer trials with GLM *β*>0.2 |  | 20 | 0 |
| Deoxygenation | fNIRS | Percentage of transfer trials with GLM *β*<‑0.2 |  | 20 | 0 |
| *Sociodemographics* | | | |  |  |
| Age | Questionnaire | Years | t0 | 47 | 0 |
| Sex | Questionnaire | Male, female | t0 | 47 | 0 |
| Years of school education | Questionnaire | <12, ≥12 | t0 | 47 | 0 |
| Professional situation | Questionnaire | Working full-time, working part-time, occasionally employed, not working | t0 | 47 | 0 |
| Therapy expectations |  |  |  |  |  |
| Motivation to change eating behavior | Questionnaire | 10-point Likert scale ranging from 1=“not at all” to 10=“completely” | t0 | 46 | 0 |
| Motivation to maintain change long term | Questionnaire | 10-point Likert scale ranging from 1=“not at all” to 10=“completely” | t0 | 46 | 0 |
| Confidence to maintain change long term | Questionnaire | 10-point Likert scale ranging from 1=“not at all” to 10=“completely” | t0 | 46 | 0 |

*Notes.* EDE=Eating Disorder Examination (Hilbert & Tuschen-Caffier, 2016a), OBE=objective binge-eating episode, EDE-Q=Eating Disorder Examination-Questionnaire (Hilbert & Tuschen-Caffier, 2016b), FCQ-T-r=Food Cravings Questionnaire-Trait Reduced (Meule et al., 2014), PHQ-9=Patient Health Questionnaire Depression Scale (Löwe et al., 2004), GAD-7=Generalized Anxiety Disorder 7 (Löwe et al., 2008), SF-12=Short Form Health Survey (Gandek et al., 1998), BIS/BAS=Behavioral Inhibition System/Behavioral Activation System Questionnaire (Strobel et al., 2006), DERS=Difficulties in Emotion Regulation Scale (Gutzweiler & In-Albon, 2018), GSES=Generalized Self-Efficacy Scale (Jerusalem & Schwarzer, 2003), PSQI=Pittsburgh Sleep Quality Index (Backhaus et al., 2002), PSS‑10=Perceived Stress Scale (Klein et al., 2016), high beta=23–28Hz, EEG=electroencephalography, fNIRS=functional near-infrared spectroscopy, GLM=generalized linear model, *β*=regression parameter in the applied general linear models representing the change in oxyhemoglobin levels.

**Table S2**

*Overview of linear models examining pretreatment EEG activity, pretreatment fNIRS activity, and rapid response as predictors of treatment outcomes for food-specific fNIRS and EEG neurofeedback*

| Predictors | Outcomes at t1 and t2 | Linear models |
| --- | --- | --- |
| Mean fronto-central high beta power during passive-viewing trials, regulation trials, and transfer trials at pretreatment (EEG); changes in prefrontal oxyhemoglobin levels during passive-viewing trials, regulation trials, and transfer trials at pretreatment (fNIRS); rapid response | Abstinence from binge eating  OBE frequency (past 28 days)  Eating disorder psychopathology  Food cravings  Body mass index  Waist-to-hip ratio  Depressive symptoms  Anxiety symptoms  Quality of life (mental and physical) | Bayesian logistic regressions  Bayesian general linear models^a^  Bayesian general linear models^b^  Bayesian general linear models^a^  Bayesian linear regressions  Bayesian linear regressions  Bayesian general linear models^a^  Bayesian general linear models^a^  Bayesian general linear models^b^ |

*Notes:* EEG=electroencephalography, fNIRS=functional near-infrared spectroscopy, high beta=23–28Hz, OBE=objective binge-eating episode.

^a^ modeled as negative binomial regression with a log link (if θ>20) or Poisson regression (if θ≤20).

^b^ modeled as beta regressions with a logit link.

**Table S3**

*Spearman correlations (ρ) between pretreatment EEG activity and baseline measures*

| Construct | Passive-viewing high beta power | Regulation high beta power | Transfer high beta power |
| --- | --- | --- | --- |
| Age (years) | .38 | .18 | .09 |
| Sex (female) | -.24 | .11 | .18 |
| Education (≥12 years) | -.24 | -.35 | -.36 |
| Illness duration (months) | -.22 | -.08 | -.18 |
| OBE frequency (EDE) | -.14 | .04 | -.25 |
| Eating disorder psychopathology (EDE‑Q) | -.17 | .26 | .27 |
| Food cravings (FCQ-T-r) | -.27 | .15 | -.07 |
| Body mass index (kg/m^2^) | .18 | .21 | .05 |
| Waist-to-hip ratio | .11 | -.09 | -.15 |
| Depressive symptoms (PHQ-9) | -.19 | .41 | .50 |
| Anxiety symptoms (GAD‑7) | -.21 | .45 | .50 |
| Mental quality of life (SF‑12) | .10 | -.49 | -.42 |
| Physical quality of life (SF‑12) | .24 | -.15 | -.09 |
| Impulsivity (BIS/BAS) |  |  |  |
| BIS | -.24 | .36 | .51 |
| BAS | -.18 | .12 | .31 |
| Difficulties in emotion regulation (DERS) | -.35 | .20 | .25 |
| Self-efficacy (GSES) | .16 | -.33 | -.38 |
| Sleep quality (PSQI) | -.06 | .57 | .59 |
| Perceived stress (PSS-10) | -.26 | .30 | .29 |
| Motivation to change eating behavior (Likert scales) | .00 | -.15 | .00 |
| Motivation to maintain change long term (Likert scales) | -.22 | -.30 | -.24 |
| Confidence to maintain change long term (Likert scales) | -.13 | -.15 | -.11 |
| Passive-viewing high beta power | 1.00 | .50 | .35 |
| Regulation high beta power | .50 | 1.00 | .85 |
| Transfer high beta power | .35 | .85 | 1.00 |

*Notes.* High beta=23–28Hz, OBE=objective binge-eating episode, EDE=Eating Disorder Examination (Hilbert & Tuschen-Caffier, 2016a), EDE-Q=Eating Disorder Examination-Questionnaire (Hilbert & Tuschen-Caffier, 2016b), FCQ-T-r=Food Cravings Questionnaire-Trait Reduced (Meule et al., 2014), PHQ-9=Patient Health Questionnaire Depression Scale (Löwe et al., 2004), GAD-7=Generalized Anxiety Disorder 7 (Löwe et al., 2008), SF-12=Short Form Health Survey (Gandek et al., 1998), BIS/BAS=Behavioral Inhibition System/Behavioral Activation System Questionnaire (Strobel et al., 2006), DERS=Difficulties in Emotion Regulation Scale (Gutzweiler & In-Albon, 2018), GSES=Generalized Self-Efficacy Scale (Jerusalem & Schwarzer, 2003), PSQI=Pittsburgh Sleep Quality Index (Backhaus et al., 2002), PSS‑10=Perceived Stress Scale (Klein et al., 2016).

**Table S4**

*Spearman correlations (ρ) between pretreatment fNIRS activity and baseline measures*

| Construct | Passive-viewing oxygenation | Passive-viewing deoxygenation | Regulation oxygenation | Regulation deoxygenation | Transfer oxygenation | Transfer deoxygenation |
| --- | --- | --- | --- | --- | --- | --- |
| Age (years) | .19 | -.15 | -.01 | -08 | -.28 | .12 |
| Sex (female) | .14 | -.23 | -.08 | -.01 | .40 | -.10 |
| Education (≥12 years) | -.11 | .19 | .10 | -.04 | -.09 | .28 |
| Illness duration (months) | .02 | -.06 | -.27 | .38 | -.59 | .35 |
| OBE frequency (EDE) | -.26 | .24 | .05 | .22 | -.15 | .28 |
| Eating disorder psychopathology (EDE‑Q) | -.06 | .02 | -.07 | .18 | .17 | -.01 |
| Food cravings (FCQ-T-r) | -.47 | .36 | .17 | -.08 | -.26 | .30 |
| Body mass index (kg/m^2^) | -.15 | .15 | .06 | .11 | -.36 | .47 |
| Waist-to-hip ratio | -.02 | .06 | -.26 | .27 | -.55 | .36 |
| Depressive symptoms (PHQ-9) | -.36 | .30 | .25 | -.20 | .10 | -.13 |
| Anxiety symptoms (GAD‑7) | -.08 | -.05 | .10 | -.03 | -.01 | -.02 |
| Mental quality of life (SF‑12) | .27 | -.15 | -.02 | .00 | .18 | -.12 |
| Physical quality of life (SF‑12) | -.19 | .11 | -.15 | .09 | .18 | -.12 |
| Impulsivity (BIS/BAS) |  |  |  |  |  |  |
| BIS | .24 | -.28 | -.25 | .26 | -.32 | .22 |
| BAS | .13 | -.14 | -.25 | .31 | -.21 | .21 |
| Difficulties in emotion regulation (DERS) | -.22 | .16 | .05 | .02 | -.35 | .19 |
| Self-efficacy (GSES) | -.12 | -.12 | -.10 | .01 | -.27 | .09 |
| Sleep quality (PSQI) | -.03 | -.05 | .11 | -.09 | .12 | -.21 |
| Perceived stress (PSS-10) | -.11 | .03 | .08 | -.09 | .06 | -.03 |
| Motivation to change eating behavior (Likert scales) | -.12 | .04 | -.18 | .16 | .16 | -.09 |
| Motivation to maintain change long term (Likert scales) | -.10 | .02 | -.12 | .04 | .04 | .02 |
| Confidence to maintain change long term (Likert scales) | -.07 | .05 | .10 | .02 | .02 | .18 |
| Passive-viewing oxygenation | 1.00 | -.90 | -.60 | .53 | -.15 | -.04 |
| Passive-viewing deoxygenation | -.90 | 1.00 | .54 | -.44 | .09 | .12 |
| Regulation oxygenation | -.60 | .54 | 1.00 | -.94 | .48 | -.20 |
| Regulation deoxygenation | -.53 | -.44 | -.94 | 1.00 | -.53 | .36 |
| Transfer oxygenation | -.15 | .09 | .48 | -.53 | 1.00 | -.74 |
| Transfer deoxygenation | -.04 | .12 | -.20 | .36 | -.74 | 1.00 |

*Notes.* oxygenation=percentage of trials with increasing oxyhemoglobin levels (*β*>0.2), deoxygenation=percentage of trials with decreasing oxyhemoglobin levels (*β*<-0.2), *β*=regression parameter in the applied general linear models representing the change in oxyhemoglobin levels, OBE=objective binge-eating episode, EDE=Eating Disorder Examination (Hilbert & Tuschen-Caffier, 2016a), EDE-Q=Eating Disorder Examination-Questionnaire (Hilbert & Tuschen-Caffier, 2016b), FCQ-T-r=Food Cravings Questionnaire-Trait Reduced (Meule et al., 2014), PHQ-9=Patient Health Questionnaire Depression Scale (Löwe et al., 2004), GAD-7=Generalized Anxiety Disorder 7 (Löwe et al., 2008), SF-12=Short Form Health Survey (Gandek et al., 1998), BIS/BAS=Behavioral Inhibition System/Behavioral Activation System Questionnaire (Strobel et al., 2006), DERS=Difficulties in Emotion Regulation Scale (Gutzweiler & In-Albon, 2018), GSES=Generalized Self-Efficacy Scale (Jerusalem & Schwarzer, 2003), PSQI=Pittsburgh Sleep Quality Index (Backhaus et al., 2002), PSS‑10=Perceived Stress Scale (Klein et al., 2016).

**Table S5**

*Partial Spearman correlations (ρ) between change in neurophysiological activity first versus last attended neurofeedback session and posttreatment abstinence from binge eating, controlling for first-session neurophysiological measures and baseline objective binge-eating frequency*

| First versus last neurofeedback session change | Abstinence from binge eating t1 |
| --- | --- |
| *EEG activity* |  |
| Δ passive-viewing high beta power (*n*=11) | -.21 |
| Δ transfer high beta power (*n*=21) | .41 |
| *fNIRS activity* |  |
| Passive-viewing trials |  |
| Δ oxygenation (*n*=19) | -.45 |
| Δ deoxygenation (*n*=19) | .03 |
| Transfer trials |  |
| Δ oxygenation (*n*=19) | .03 |
| Δ deoxygenation (*n*=19) | -.24 |

*Notes*. high beta=23–28Hz, oxygenation=percentage of trials with increasing oxyhemoglobin levels (*β*>0.2), deoxygenation=percentage of trials with decreasing oxyhemoglobin levels (*β*<-0.2), *β*=regression parameter in the applied general linear models representing the change in oxyhemoglobin levels.

**Table S6**

*Results from the Bayesian linear models assessing pretreatment EEG activity as predictors of secondary outcomes (n=19)*

|  | Regression coefficient (*β*) | | | |  | Bayesian *R*^2^ or Δ*R*^2^ | | | |
| --- | --- | --- | --- | --- | --- | --- | --- | --- | --- |
| Model and predictors | *M* | *SD* | 95% CrI | |  | *M* | *SD* | 95% CrI | |
|  |  |  | lower | upper | |  |  | lower | upper |
| Eating disorder psychopathology (EDE-Q) t1 |  |  |  |  | | 0.43 | 0.11 | 0.21 | 0.64 |
| Intercept | -0.07 | 0.15 | -0.37 | 0.23 | |  |  |  |  |
| Eating disorder psychopathology (EDE-Q) t0 | 0.74 | 0.17 | 0.40 | 1.08 | | 0.40 | 0.09 | 0.23 | 0.57 |
| Transfer high beta power (EEG) | -0.15 | 0.14 | -0.43 | 0.12 | | 0.02 | 0.12 | <0.01 | 0.24 |
| Eating disorder psychopathology (EDE-Q) t2 |  |  |  |  | | 0.22 | 0.14 | <0.01 | 0.50 |
| Intercept | -0.08 | 0.18 | -0.43 | 0.27 | |  |  |  |  |
| Eating disorder psychopathology (EDE-Q) t0^b^ | 0.44 | 0.23 | 0.00 | 0.88 | | 0.15 | 0.11 | <0.01 | 0.37 |
| Passive-viewing high beta power (EEG) | -0.23 | 0.19 | -0.60 | 0.13 | | 0.04 | 0.13 | <0.01 | 0.29 |
| Food cravings (FCQ-T-r) t1 |  |  |  |  | | 0.56 | 0.07 | 0.43 | 0.70 |
| Intercept | 3.95 | 0.03 | 3.89 | 4.01 | |  |  |  |  |
| Food cravings (FCQ-T-r) t0 | 0.12 | 0.03 | 0.07 | 0.18 | | 0.18 | 0.10 | <0.01 | 0.37 |
| Regulation high beta power (EEG) | 0.14 | 0.04 | 0.06 | 0.22 | | 0.10 | 0.09 | <0.01 | 0.28 |
| Transfer high beta power (EEG) | -0.23 | 0.04 | -0.30 | -0.16 | | 0.28 | 0.09 | 0.11 | 0.46 |
| Food cravings (FCQ-T-r) t2 |  |  |  |  | | 0.32 | 0.07 | 0.19 | 0.45 |
| Intercept | 3.93 | 0.03 | 3.86 | 3.99 | |  |  |  |  |
| Food cravings (FCQ-T-r) t0 | 0.05 | 0.05 | -0.04 | 0.14 | | 0.05 | 0.09 | <0.01 | 0.23 |
| Regulation high beta power (EEG) | 0.15 | 0.04 | 0.07 | 0.23 | | 0.10 | 0.10 | <0.01 | 0.29 |
| Transfer high beta power (EEG) | -0.22 | 0.06 | -0.34 | -0.12 | | 0.23 | 0.08 | 0.07 | 0.39 |
| Body mass index (kg/m^2^) t1 |  |  |  |  | | 0.98 | 0.00 | 0.97 | 0.98 |
| Intercept | 0.00 | 0.03 | -0.05 | 0.05 | |  |  |  |  |
| Body mass index (kg/m^2^) t0 | 0.97 | 0.03 | 0.92 | 1.02 | | 0.83 | 0.09 | 0.64 | 0.96 |
| Regulation high beta power (EEG) | 0.12 | 0.04 | 0.04 | 0.19 | | <0.01 | 0.00 | <0.01 | 0.01 |
| Transfer high beta power (EEG)^a^ | -0.11 | 0.04 | -0.19 | -0.03 | | <0.01 | 0.00 | <0.01 | 0.01 |
| Body mass index (kg/m^2^) t2 |  |  |  |  | | 0.75 | 0.14 | 0.48 | 1.00 |
| Intercept | 0.00 | 0.08 | -0.16 | 0.16 | |  |  |  |  |
| Body mass index (kg/m^2^) t0 | 0.87 | 0.11 | 0.64 | 1.09 | | 0.71 | 0.07 | 0.59 | 0.84 |
| Passive-viewing high beta power (EEG)^b^ | -0.16 | 0.10 | -0.35 | 0.04 | | 0.02 | 0.08 | <0.01 | 0.17 |
| Waist-to-hip ratio t1 |  |  |  |  | | 0.46 | 0.23 | 0.01 | 0.91 |
| Intercept | 0.00 | 0.13 | -0.25 | 0.25 | |  |  |  |  |
| Waist-to-hip ratio t0 | 0.68 | 0.23 | 0.22 | 1.13 | | 0.43 | 0.10 | 0.23 | 0.62 |
| Transfer high beta power (EEG) | 0.20 | 0.15 | -0.09 | 0.50 | | 0.04 | 0.13 | <0.01 | 0.30 |
| Waist-to-hip ratio t2 |  |  |  |  | | 0.55 | 0.21 | 0.14 | 0.96 |
| Intercept | 0.00 | 0.11 | -0.22 | 0.22 | |  |  |  |  |
| Waist-to-hip ratio t0 | 0.71 | 0.18 | 0.37 | 1.06 | | 0.46 | 0.12 | 0.24 | 0.69 |
| Transfer high beta power (EEG) | -0.07 | 0.16 | -0.38 | 0.23 | | 0.01 | 0.12 | <0.01 | 0.25 |
| Depressive symptoms (PHQ-9) t1 |  |  |  |  | | 0.65 | 0.09 | 0.48 | 0.82 |
| Intercept | 1.92 | 0.07 | 1.78 | 2.06 | |  |  |  |  |
| Depressive symptoms (PHQ-9) t0 | 0.49 | 0.07 | 0.35 | 0.62 | | 0.59 | 0.10 | 0.38 | 0.79 |
| Passive-viewing high beta power (EEG)^b^ | 0.14 | 0.07 | 0.00 | 0.27 | | 0.05 | 0.13 | <0.01 | 0.30 |
| Transfer high beta power (EEG)^a^ | -0.19 | 0.08 | -0.34 | -0.04 | | 0.10 | 0.13 | <0.01 | 0.36 |
| Depressive symptoms (PHQ-9) t2 |  |  |  |  | | 0.35 | 0.14 | 0.08 | 0.62 |
| Intercept | 1.99 | 0.07 | 1.85 | 2.13 | |  |  |  |  |
| Depressive symptoms (PHQ-9) t0 | 0.25 | 0.08 | 0.10 | 0.40 | | 0.32 | 0.13 | 0.07 | 0.58 |
| Transfer high beta power (EEG)^b^ | -0.14 | 0.07 | -0.29 | 0.00 | | 0.12 | 0.17 | <0.01 | 0.45 |
| Anxiety symptoms (GAD-7) t1 |  |  |  |  | | 0.54 | 0.11 | 0.33 | 0.74 |
| Intercept | 1.38 | 0.09 | 1.22 | 1.55 | |  |  |  |  |
| Anxiety symptoms (GAD-7) t0 | 0.61 | 0.09 | 0.44 | 0.78 | | 0.46 | 0.11 | 0.24 | 0.68 |
| Transfer high beta power (EEG)^b^ | -0.16 | 0.08 | -0.33 | 0.00 | | 0.02 | 0.12 | <0.01 | 0.25 |
| Anxiety symptoms (GAD-7) t2 |  |  |  |  | | 0.25 | 0.15 | <0.01 | 0.54 |
| Intercept | 1.69 | 0.11 | 1.47 | 1.91 | |  |  |  |  |
| Anxiety symptoms (GAD-7) t0^a^ | 0.27 | 0.13 | 0.01 | 0.53 | | 0.18 | 0.14 | <0.01 | 0.45 |
| Regulation high beta power (EEG) | 0.08 | 0.10 | -0.12 | 0.28 | | 0.02 | 0.16 | <0.01 | 0.33 |
| Mental quality of life (SF-12) t1 |  |  |  |  | | 0.38 | 0.14 | 0.11 | 0.65 |
| Intercept | 0.09 | 0.18 | -0.27 | 0.46 | |  |  |  |  |
| Mental quality of life (SF-12) t0 | 0.89 | 0.25 | 0.41 | 1.37 | | 0.35 | 0.10 | 0.16 | 0.54 |
| Regulation high beta power (EEG) | 0.12 | 0.20 | -0.27 | 0.51 | | 0.02 | 0.13 | <0.01 | 0.27 |
| Mental quality of life (SF-12) t2 |  |  |  |  | | 0.37 | 0.14 | 0.09 | 0.65 |
| Intercept | 0.08 | 0.17 | -0.24 | 0.41 | |  |  |  |  |
| Mental quality of life (SF-12) t0^a^ | 0.52 | 0.23 | 0.07 | 0.97 | | 0.18 | 0.13 | <0.01 | 0.42 |
| Regulation high beta power (EEG)^a^ | -0.43 | 0.18 | -0.77 | -0.08 | | 0.12 | 0.13 | <0.01 | 0.37 |
| Physical quality of life (SF-12) t1 |  |  |  |  | | 0.34 | 0.20 | <0.01 | 0.72 |
| Intercept | 0.60 | 0.21 | 0.20 | 1.01 | |  |  |  |  |
| Physical quality of life (SF-12) t0 | 0.73 | 0.33 | 0.09 | 1.37 | | 0.31 | 0.09 | 0.13 | 0.49 |
| Transfer high beta power (EEG) | 0.12 | 0.20 | -0.26 | 0.51 | | 0.02 | 0.12 | <0.01 | 0.27 |
| Physical quality of life (SF-12) t2 |  |  |  |  | | 0.16 | 0.11 | <0.01 | 0.39 |
| Intercept | 0.23 | 0.20 | -0.17 | 0.63 | |  |  |  |  |
| Physical quality of life (SF-12) t0^b^ | 0.40 | 0.23 | -0.05 | 0.85 | | 0.12 | 0.10 | <0.01 | 0.31 |
| Transfer high beta power (EEG) | 0.27 | 0.24 | -0.20 | 0.74 | | 0.08 | 0.11 | <0.01 | 0.29 |

*Notes.* CrI=credible interval, high beta=23–28Hz, EDE-Q=Eating Disorder Examination-Questionnaire (Hilbert & Tuschen-Caffier, 2016b), FCQ-T-r=Food Cravings Questionnaire-Trait Reduced (Meule et al., 2014), PHQ-9=Patient Health Questionnaire Depression Scale (Löwe et al., 2004), GAD-7=Generalized Anxiety Disorder 7 (Löwe et al., 2008), SF-12=Short Form Health Survey (Gandek et al., 1998).

^a^ 95% CrI did include 0 in the sensitivity analysis with doubled prior standard deviations.

^b^ 95% CrI did not longer include 0 in the sensitivity analysis with halved prior standard deviations.

**Table S7**

*Results from the Bayesian linear models assessing pretreatment fNIRS activity as predictors of secondary outcomes (n=20)*

|  | | Regression coefficient (*β*) | | | | |  | | Bayesian *R*^2^ or Δ*R*^2^ | | | | | | |
| --- | --- | --- | --- | --- | --- | --- | --- | --- | --- | --- | --- | --- | --- | --- | --- |
| Model and predictors | | *M* | *SD* | 95% CrI | | |  | | *M* | *SD* | | 95% CrI | | | |
|  | |  |  | lower | upper | | | |  |  | | lower | | upper | |
| Eating disorder psychopathology (EDE-Q) t1 |  |  |  | |  | | 0.47 | | | 0.11 | 0.25 | 0.70 | |  |  |
| Intercept | 0.13 | 0.14 | -0.14 | | 0.40 | |  | | |  |  |  | |  |  |
| Eating disorder psychopathology (EDE-Q) t0 | 0.62 | 0.20 | 0.22 | | 1.02 | | 0.26 | | | 0.15 | <0.01 | 0.57 | |  |  |
| Passive-viewing oxygenation (fNIRS)^a^ | -0.35 | 0.17 | -0.67 | | -0.03 | | 0.07 | | | 0.13 | <0.01 | 0.32 | |  |  |
| Eating disorder psychopathology (EDE-Q) t2 |  |  |  | |  | | 0.18 | | | 0.13 | <0.01 | 0.44 | |  |  |
| Intercept | 0.25 | 0.16 | -0.06 | | 0.56 | |  | | |  |  |  | |  |  |
| Eating disorder psychopathology (EDE-Q) t0 | 0.40 | 0.25 | -0.08 | | 0.88 | | 0.14 | | | 0.10 | <0.01 | 0.33 | |  |  |
| Regulation oxygenation (fNIRS) | -0.21 | 0.18 | -0.58 | | 0.15 | | 0.06 | | | 0.11 | <0.01 | 0.28 | |  |  |
| Food cravings (FCQ-T-r) t1 |  |  |  | |  | | 0.47 | | | 0.08 | 0.32 | 0.62 | |  |  |
| Intercept | 3.84 | 0.02 | 3.80 | | 3.89 | |  | | |  |  |  | |  |  |
| Food cravings (FCQ-T-r) t0 | 0.12 | 0.03 | 0.06 | | 0.17 | | 0.18 | | | 0.13 | <0.01 | 0.43 | |  |  |
| Passive-viewing deoxygenation (fNIRS) | 0.10 | 0.02 | 0.05 | | 0.15 | | 0.14 | | | 0.11 | <0.01 | 0.36 | |  |  |
| Food cravings (FCQ-T-r) t2 |  |  |  | |  | | 0.20 | | | 0.09 | 0.01 | 0.38 | |  |  |
| Intercept | 3.84 | 0.05 | 3.74 | | 3.93 | |  | | |  |  |  | |  |  |
| Food cravings (FCQ-T-r) t0^a^ | 0.12 | 0.06 | 0.02 | | 0.23 | | 0.14 | | | 0.12 | <0.01 | 0.37 | |  |  |
| Trans oxygenation (fNIRS) | 0.08 | 0.06 | -0.03 | | 0.19 | | 0.08 | | | 0.13 | <0.01 | 0.33 | |  |  |
| Body mass index (kg/m2) t1 |  |  |  | |  | | 0.96 | | | 0.00 | 0.95 | 0.96 | |  |  |
| Intercept | 0.00 | 0.03 | -0.07 | | 0.07 | |  | | |  |  |  | |  |  |
| Body mass index (kg/m2) t0 | 0.97 | 0.03 | 0.90 | | 1.04 | | 0.90 | | | 0.06 | 0.76 | 0.96 | |  |  |
| Regulation oxygenation (fNIRS) | 0.05 | 0.04 | -0.02 | | 0.12 | | <0.01 | | | 0.01 | <0.01 | 0.01 | |  |  |
| Body mass index (kg/m2) t2 |  |  |  | |  | | 0.60 | | | 0.13 | 0.34 | 0.87 | |  |  |
| Intercept | 0.00 | 0.10 | -0.20 | | 0.20 | |  | | |  |  |  | |  |  |
| Body mass index (kg/m^2^) t0 | 0.80 | 0.13 | 0.54 | | 1.06 | | 0.58 | | | 0.08 | 0.41 | 0.74 | |  |  |
| Passive-viewing deoxygenation (fNIRS) | -0.14 | 0.11 | -0.36 | | 0.07 | | 0.02 | | | 0.11 | <0.01 | 0.23 | |  |  |
| Waist-to-hip ratio t1 |  |  |  | |  | | 0.49 | | | 0.13 | 0.23 | 0.76 | |  |  |
| Intercept | 0.00 | 0.12 | -0.23 | | 0.24 | |  | | |  |  |  | |  |  |
| Waist-to-hip ratio t0^a^ | 0.41 | 0.18 | 0.05 | | 0.77 | | 0.12 | | | 0.15 | <0.01 | 0.41 | |  |  |
| Trans oxygenation (fNIRS) | -0.57 | 0.21 | -0.98 | | -0.16 | | 0.11 | | | 0.15 | <0.01 | 0.40 | |  |  |
| Trans deoxygenation (fNIRS) | -0.33 | 0.25 | -0.81 | | 0.16 | | 0.05 | | | 0.15 | <0.01 | 0.35 | |  |  |
| Waist-to-hip ratio t2 |  |  |  | |  | | 0.48 | | | 0.16 | 0.17 | 0.78 | |  |  |
| Intercept | 0.00 | 0.12 | -0.23 | | 0.23 | |  | | |  |  |  | |  |  |
| Waist-to-hip ratio t0 | 0.47 | 0.28 | -0.07 | | 1.01 | | 0.25 | | | 0.13 | <0.01 | 0.51 | |  |  |
| Passive-viewing oxygenation (fNIRS)^a^ | 0.50 | 0.24 | 0.02 | | 0.97 | | 0.26 | | | 0.13 | 0.01 | 0.51 | |  |  |
| Depressive symptoms (PHQ-9) t1 |  |  |  | |  | | 0.25 | | | 0.13 | <0.01 | 0.50 | |  |  |
| Intercept | 2.08 | 0.06 | 1.97 | | 2.19 | |  | | |  |  |  | |  |  |
| Depressive symptoms (PHQ-9) t0^a^ | 0.16 | 0.07 | 0.01 | | 0.30 | | 0.16 | | | 0.15 | <0.01 | 0.45 | |  |  |
| Regulation oxygenation (fNIRS) | 0.09 | 0.06 | -0.03 | | 0.21 | | 0.05 | | | 0.15 | <0.01 | 0.34 | |  |  |
| Depressive symptoms (PHQ-9) t2 |  |  |  | |  | | 0.10 | | | 0.08 | <0.01 | 0.26 | |  |  |
| Intercept | 2.19 | 0.07 | 2.04 | | 2.34 | |  | | |  |  |  | |  |  |
| Depressive symptoms (PHQ-9) t0 | 0.01 | 0.18 | -0.34 | | 0.36 | | 0.07 | | | 0.09 | <0.01 | 0.25 | |  |  |
| Passive-viewing oxygenation (fNIRS) | -0.05 | 0.10 | -0.25 | | 0.14 | | 0.03 | | | 0.10 | <0.01 | 0.24 | |  |  |
| Anxiety symptoms (GAD-7) t1 |  |  |  | |  | | 0.39 | | | 0.11 | 0.17 | 0.60 | |  |  |
| Intercept | 1.44 | 0.08 | 1.28 | | 1.60 | |  | | |  |  |  | |  |  |
| Anxiety symptoms (GAD-7) t0^a^ | 0.22 | 0.10 | 0.01 | | 0.42 | | 0.13 | | | 0.16 | <0.01 | 0.43 | |  |  |
| Transfer oxygenation (fNIRS) | -0.38 | 0.09 | -0.55 | | -0.21 | | 0.23 | | | 0.13 | <0.01 | 0.49 | |  |  |
| Anxiety symptoms (GAD-7) t2 |  |  |  | |  | | 0.16 | | | 0.14 | <0.01 | 0.45 | |  |  |
| Intercept | 1.79 | 0.12 | 1.56 | | 2.02 | |  | | |  |  |  | |  |  |
| Anxiety symptoms (GAD-7) t0 | 0.15 | 0.22 | -0.28 | | 0.59 | | 0.10 | | | 0.12 | <0.01 | 0.33 | |  |  |
| Passive-viewing deoxygenation (fNIRS)^b^ | -0.16 | 0.12 | -0.39 | | 0.08 | | 0.06 | | | 0.12 | <0.01 | 0.30 | |  |  |
| Mental quality of life (SF-12) t1 |  |  |  | |  | | 0.33 | | | 0.10 | 0.14 | 0.52 | |  |  |
| Intercept | 0.21 | 0.16 | -0.09 | | 0.52 | |  | | |  |  |  | |  |  |
| Mental quality of life (SF-12) t0 | 0.22 | 0.21 | -0.19 | | 0.64 | | 0.05 | | | 0.15 | <0.01 | 0.35 | |  |  |
| Passive-viewing oxygenation (fNIRS)^a^ | 0.49 | 0.18 | 0.13 | | 0.85 | | 0.12 | | | 0.13 | <0.01 | 0.38 | |  |  |
| Transfer deoxygenation (fNIRS)^a^ | -0.39 | 0.16 | -0.70 | | -0.08 | | 0.09 | | | 0.15 | <0.01 | 0.37 | |  |  |
| Mental quality of life (SF-12) t2 |  |  |  | |  | | 0.45 | | | 0.17 | 0.11 | 0.78 | |  |  |
| Intercept | 0.52 | 0.16 | 0.20 | | 0.85 | |  | | |  |  |  | |  |  |
| Mental quality of life (SF-12) t0 | 0.55 | 0.33 | -0.09 | | 1.20 | | 0.20 | | | 0.12 | <0.01 | 0.44 | |  |  |
| Passive-viewing oxygenation (fNIRS)^b^ | 0.64 | 0.37 | -0.08 | | 1.36 | | 0.10 | | | 0.12 | <0.01 | 0.34 | |  |  |
| Passive-viewing deoxygenation (fNIRS) | 0.87 | 0.30 | 0.27 | | 1.46 | | 0.18 | | | 0.12 | <0.01 | 0.42 | |  |  |
| Physical quality of life (SF-12) t1 |  |  |  | |  | | 0.54 | | | 0.08 | 0.38 | 0.69 | |  |  |
| Intercept | 0.06 | 0.14 | -0.22 | | 0.35 | |  | | |  |  |  | |  |  |
| Physical quality of life (SF-12) t0 | 1.03 | 0.19 | 0.67 | | 1.40 | | 0.44 | | | 0.11 | 0.23 | 0.65 | |  |  |
| Passive-viewing oxygenation (fNIRS)^a^ | 0.43 | 0.19 | 0.05 | | 0.81 | | 0.07 | | | 0.11 | <0.01 | 0.30 | |  |  |
| Regulation oxygenation (fNIRS)^a^ | 0.37 | 0.19 | 0.01 | | 0.73 | | 0.08 | | | 0.11 | <0.01 | 0.30 | |  |  |
| Transfer oxygenation (fNIRS)^a^ | -0.64 | 0.22 | -1.06 | | -0.21 | | 0.07 | | | 0.12 | <0.01 | 0.31 | |  |  |
| Transfer deoxygenation (fNIRS)^a^ | -0.45 | 0.22 | -0.88 | | -0.01 | | 0.03 | | | 0.11 | <0.01 | 0.25 | |  |  |
| Physical quality of life (SF-12) t2 |  |  |  | |  | | 0.27 | | | 0.13 | 0.02 | 0.51 | |  |  |
| Intercept | -0.04 | 0.17 | -0.38 | | 0.30 | |  | | |  |  |  | |  |  |
| Physical quality of life (SF-12) t0 | 0.62 | 0.22 | 0.18 | | 1.06 | | 0.24 | | | 0.10 | 0.04 | 0.43 | |  |  |
| Passive-viewing deoxygenation (fNIRS)^b^ | -0.29 | 0.19 | -0.66 | | 0.07 | | 0.05 | | | 0.14 | <0.01 | 0.32 | |  |  |

*Notes.* CrI=credible interval, oxygenation=percentage of trials with increasing oxyhemoglobin levels (*β*>0.2), deoxygenation=percentage of trials with decreasing oxyhemoglobin levels (*β*<-0.2), *β*=regression parameter in the applied general linear models representing the change in oxyhemoglobin levels, EDE-Q=Eating Disorder Examination-Questionnaire (Hilbert & Tuschen-Caffier, 2016b), FCQ-T-r=Food Cravings Questionnaire-Trait Reduced (Meule et al., 2014), PHQ-9=Patient Health Questionnaire Depression Scale (Löwe et al., 2004), GAD-7=Generalized Anxiety Disorder 7 (Löwe et al., 2008), SF-12=Short Form Health Survey (Gandek et al., 1998).

^a^ 95% CrI did include 0 in the sensitivity analysis with doubled prior standard deviations.

^b^ 95% CrI did not longer include 0 in the sensitivity analysis with halved prior standard deviations.

**Table S8**

*Results from the Bayesian linear models assessing rapid response as predictor of secondary outcomes (n=28)*

|  | Regression coefficient (*β*) | | | |  | Bayesian *R*^2^ or Δ*R*^2^ | | | |
| --- | --- | --- | --- | --- | --- | --- | --- | --- | --- |
| Model and predictors | *M* | *SD* | 95% CrI | |  | *M* | *SD* | 95% CrI | |
|  |  |  | lower | upper | |  |  | lower | upper |

| Eating disorder psychopathology (EDE-Q) t1 |  |  |  |  | 0.46 | 0.07 | 0.31 | 0.60 |
| --- | --- | --- | --- | --- | --- | --- | --- | --- |
| Intercept | -0.08 | 0.13 | -0.33 | 0.17 |  |  |  |  |
| Eating disorder psychopathology (EDE-Q) t0 | 0.78 | 0.15 | 0.48 | 1.08 | 0.32 | 0.10 | 0.12 | 0.52 |
| Rapid response^b^ | -0.21 | 0.14 | -0.48 | 0.06 | 0.03 | 0.09 | <0.01 | 0.21 |
| Eating disorder psychopathology (EDE-Q) t2 |  |  |  |  | 0.25 | 0.09 | 0.07 | 0.43 |
| Intercept | -0.11 | 0.13 | -0.37 | 0.15 |  |  |  |  |
| Eating disorder psychopathology (EDE-Q) t0 | 0.31 | 0.19 | -0.06 | 0.67 | 0.09 | 0.12 | <0.01 | 0.31 |
| Rapid response^a^ | -0.37 | 0.16 | -0.69 | -0.05 | 0.11 | 0.11 | <0.01 | 0.33 |
| Food cravings (FCQ-T-r) t1 |  |  |  |  | 0.33 | 0.06 | 0.20 | 0.45 |
| Intercept | 3.90 | 0.02 | 3.86 | 3.94 |  |  |  |  |
| Food cravings (FCQ-T-r) t0 | 0.08 | 0.02 | 0.04 | 0.12 | 0.09 | 0.09 | <0.01 | 0.25 |
| Rapid response | -0.13 | 0.02 | -0.17 | -0.09 | 0.22 | 0.08 | 0.07 | 0.37 |
| Food cravings (FCQ-T-r) t2 |  |  |  |  | 0.15 | 0.07 | 0.01 | 0.29 |
| Intercept | 3.89 | 0.04 | 3.82 | 3.96 |  |  |  |  |
| Food cravings (FCQ-T-r) t0 | 0.03 | 0.05 | -0.06 | 0.12 | 0.03 | 0.11 | <0.01 | 0.24 |
| Rapid response^a^ | -0.10 | 0.04 | -0.18 | -0.02 | 0.11 | 0.09 | <0.01 | 0.28 |
| Body mass index (kg/m^2^) t1 |  |  |  |  | 0.95 | 0.00 | 0.94 | 0.96 |
| Intercept | 0.00 | 0.03 | -0.06 | 0.06 |  |  |  |  |
| Body mass index (kg/m^2^) t0 | 1.00 | 0.03 | 0.94 | 1.07 | 0.84 | 0.07 | 0.70 | 0.95 |
| Rapid response^a^ | 0.08 | 0.03 | 0.01 | 0.14 | <0.01 | 0.01 | <0.01 | 0.01 |
| Body mass index (kg/m^2^) t2 |  |  |  |  | 0.77 | 0.08 | 0.60 | 0.93 |
| Intercept | 0.00 | 0.07 | -0.13 | 0.13 |  |  |  |  |
| Body mass index (kg/m^2^) t0 | 0.92 | 0.08 | 0.76 | 1.09 | 0.68 | 0.08 | 0.53 | 0.83 |
| Neurofeedback group (EEG, rtfNIRS)^b^ | -0.17 | 0.09 | -0.35 | 0.00 | 0.03 | 0.05 | <0.01 | 0.13 |
| Rapid response^a^ | 0.22 | 0.09 | 0.04 | 0.40 | 0.04 | 0.05 | <0.01 | 0.14 |
| Waist-to-hip ratio t1 |  |  |  |  | 0.48 | 0.10 | 0.28 | 0.68 |
| Intercept | 0.00 | 0.10 | -0.19 | 0.19 |  |  |  |  |
| Waist-to-hip ratio t0 | 0.68 | 0.11 | 0.46 | 0.90 | 0.44 | 0.09 | 0.25 | 0.62 |
| Rapid response | 0.13 | 0.12 | -0.11 | 0.36 | 0.02 | 0.11 | <0.01 | 0.24 |
| Waist-to-hip ratio t2 |  |  |  |  | 0.49 | 0.18 | 0.15 | 0.83 |
| Intercept | 0.00 | 0.10 | -0.19 | 0.19 |  |  |  |  |
| Waist-to-hip ratio t0 | 0.69 | 0.17 | 0.35 | 1.03 | 0.46 | 0.08 | 0.30 | 0.62 |
| Rapid response | 0.04 | 0.14 | -0.24 | 0.32 | 0.01 | 0.11 | <0.01 | 0.22 |
| Depressive symptoms (PHQ-9) t1 |  |  |  |  | 0.36 | 0.12 | 0.12 | 0.60 |
| Intercept | 2.12 | 0.05 | 2.01 | 2.22 |  |  |  |  |
| Depressive symptoms (PHQ-9) t0 | 0.26 | 0.06 | 0.14 | 0.38 | 0.35 | 0.09 | 0.16 | 0.53 |
| Rapid response | -0.05 | 0.05 | -0.14 | 0.05 | 0.02 | 013 | <0.01 | 0.28 |
| Depressive symptoms (PHQ-9) t2 |  |  |  |  | 0.11 | 0.08 | <0.01 | 0.27 |
| Intercept | 2.21 | 0.06 | 2.09 | 2.32 |  |  |  |  |
| Depressive symptoms (PHQ-9) t0 | 0.08 | 0.12 | -0.15 | 0.31 | 0.06 | 0.08 | <0.01 | 0.22 |
| Neurofeedback group (EEG, rtfNIRS) | 0.08 | 0.06 | -0.04 | 0.20 | 0.03 | 0.09 | <0.01 | 0.20 |
| Rapid response | -0.03 | 0.07 | -0.16 | 0.11 | 0.02 | 0.09 | <0.01 | 0.19 |
| Anxiety symptoms (GAD-7) t1 |  |  |  |  | 0.50 | 0.11 | 0.29 | 0.72 |
| Intercept | 1.64 | 0.06 | 1.52 | 1.77 |  |  |  |  |
| Anxiety symptoms (GAD-7) t0 | 0.38 | 0.06 | 0.27 | 0.49 | 0.47 | 0.11 | 0.26 | 0.69 |
| Rapid response | 0.04 | 0.06 | -0.08 | 0.16 | 0.01 | 0.14 | <0.01 | 0.29 |
| Anxiety symptoms (GAD-7) t2 |  |  |  |  | 0.16 | 0.09 | <0.01 | 0.33 |
| Intercept | 1.91 | 0.08 | 1.76 | 2.07 |  |  |  |  |
| Anxiety symptoms (GAD-7) t0 | 0.21 | 0.10 | 0.01 | 0.40 | 0.11 | 0.09 | <0.01 | 0.29 |
| Neurofeedback group (EEG, rtfNIRS)^b^ | 0.12 | 0.09 | -0.06 | 0.31 | 0.04 | 0.11 | <0.01 | 0.26 |
| Rapid response | -0.07 | 0.11 | -0.30 | 0.15 | 0.03 | 0.11 | <0.01 | 0.25 |
| Mental quality of life (SF-12) t1 |  |  |  |  | 0.34 | 0.09 | 0.16 | 0.52 |
| Intercept | 0.10 | 0.15 | -0.19 | 0.38 |  |  |  |  |
| Mental quality of life (SF-12) t0 | 0.56 | 0.15 | 0.26 | 0.85 | 0.14 | 0.09 | <0.01 | 0.33 |
| Neurofeedback group (EEG, rtfNIRS) | 0.05 | 0.15 | -0.24 | 0.33 | 0.01 | 0.10 | <0.01 | 0.19 |
| Mental quality of life (SF-12) t0 x Neurofeedback group (EEG, rtfNIRS)^a^ | -0.40 | 0.17 | -0.74 | -0.07 | 0.06 | 0.10 | <0.01 | 0.26 |
| Rapid response | 0.02 | 0.14 | -0.26 | 0.30 | 0.01 | 0.09 | <0.01 | 0.19 |
| Mental quality of life (SF-12) t2 |  |  |  |  | 0.32 | 0.13 | 0.07 | 0.57 |
| Intercept | 0.35 | 0.12 | 0.12 | 0.58 |  |  |  |  |
| Mental quality of life (SF-12) t0 | 0.50 | 0.19 | 0.12 | 0.87 | 0.22 | 0.11 | 0.01 | 0.43 |
| Neurofeedback group (EEG, rtfNIRS)^a^ | -0.30 | 0.13 | -0.55 | -0.05 | 0.07 | 0.12 | <0.01 | 0.30 |
| Rapid response | 0.05 | 0.15 | -0.25 | 0.35 | 0.01 | 0.12 | <0.01 | 0.24 |
| Physical quality of life (SF-12) t1 |  |  |  |  | 0.45 | 0.15 | 0.16 | 0.74 |
| Intercept | 0.16 | 0.13 | -0.10 | 0.42 |  |  |  |  |
| Physical quality of life (SF-12) t0 | 0.75 | 0.18 | 0.41 | 1.10 | 0.43 | 0.08 | 0.27 | 0.59 |
| Rapid response | 0.07 | 0.13 | -0.19 | 0.33 | 0.01 | 0.11 | <0.01 | 0.23 |
| Physical quality of life (SF-12) t2 |  |  |  |  | 0.25 | 0.11 | 0.05 | 0.46 |
| Intercept | -0.03 | 0.14 | -0.31 | 0.25 |  |  |  |  |
| Physical quality of life (SF-12) t0^a^ | 0.35 | 0.15 | 0.05 | 0.65 | 0.13 | 0.12 | <0.01 | 0.36 |
| Rapid response^a^ | -0.35 | 0.15 | -0.66 | -0.05 | 0.11 | 0.12 | <0.01 | 0.36 |

*Notes.* CrI=credible interval, rapid response=at least 91.67% reduction in OBE frequency at week 4 of treatment, EDE-Q=Eating Disorder Examination-Questionnaire (Hilbert & Tuschen-Caffier, 2016b), FCQ-T-r=Food Cravings Questionnaire-Trait Reduced (Meule et al., 2014), PHQ-9=Patient Health Questionnaire Depression Scale (Löwe et al., 2004), GAD-7=Generalized Anxiety Disorder 7 (Löwe et al., 2008), SF-12=Short Form Health Survey (Gandek et al., 1998).

^a^ 95% CrI did include 0 in the sensitivity analysis with doubled prior standard deviations.

^b^ 95% CrI did not longer include 0 in the sensitivity analysis with halved prior standard deviations.

**Table S9**

*Cutoffs for definition of rapid response based on reduction in objective binge eating episodes from weeks 1–4*

| Timepoint | AUC Estimate | AUC 95% CI | | Sensitivity | Specificity | Cutoff |
| --- | --- | --- | --- | --- | --- | --- |
|  |  | lower | upper |  |  |  |
| Week 1 | .44 | .12 | .77 | .20 | .93 | 92.86 |
| Week 2 | .51 | .21 | .82 | .50 | .67 | 73.21 |
| Week 3 | .69 | .40 | .99 | .67 | .84 | 92.86 |
| Week 4 | .73 | .41 | 1.00 | .75 | .79 | 91.67 |

*Notes.* AUC=area under the curve, CI=confidence interval.

**Table S10**

*Additional descriptive baseline sample characteristics*

| Construct | *M* (*SD*) or *n* (%) | | Valid values (*n*) | | Imputed values (*n*) | |
| --- | --- | --- | --- | --- | --- | --- |
|  | EEG-NF | rtfNIRS-NF | EEG-NF | rtfNIRS-NF | EEG-NF | rtfNIRS-NF |
| *Therapy expectations* |  |  |  |  |  |  |
| motivation to change eating behavior (Likert scale, 1–10) | 8.88 (1.27) | 8.64 (1.67) | 24 | 22 | - | - |
| motivation to maintain change long term (Likert scale, 1–10) | 8.58 (1.69) | 8.77 (1.32) | 24 | 22 | - | - |
| confidence to maintain change long term (Likert scale, 1–10) | 6.33 (2.45) | 6.59 (1.95) | 24 | 22 | - | - |
| *General psychopathology* |  |  |  |  |  |  |
| Impulsivity (BIS/BAS) |  |  |  |  |  |  |
| BIS (1–4) | 3.14 (0.42) | 2.93 (0.53) | 23 | 22 | 1 | 1 |
| BAS (1–4) | 2.98 (0.31) | 2.91 (0.34) | 23 | 22 | 1 | 1 |
| Difficulties in emotion regulation (DERS, 26–180) | 87.63 (23.27) | 85.64 (20.12) | 23 | 22 | 1 | 1 |
| Self-efficacy (GSES, 10–40) | 26.64 (5.27) | 27.95 (4.89) | 23 | 22 | 1 | 1 |
| Sleep quality (PSQI, 0–21) | 11.48 (4.10) | 9.99 (2.81) | 21 | 22 | 3 | 1 |
| Perceived stress (PSS-10, 0–40) | 18.18 (7.35) | 17.00 (4.78) | 23 | 22 | 1 | 1 |

*Notes.* BIS/BAS=Behavioral Inhibition System/Behavioral Activation System Questionnaire (Strobel et al., 2006), DERS=Difficulties in Emotion Regulation Scale (Gutzweiler & In-Albon, 2018), GSES=Generalized Self-Efficacy Scale (Jerusalem & Schwarzer, 2003), PSQI=Pittsburgh Sleep Quality Index (Backhaus et al., 2002), PSS‑10=Perceived Stress Scale (Klein et al., 2016).

**Table S11**

*Results from the Bayesian linear models assessing baseline and pretreatment variable as predictor of rapid response*

| Parameter | *M* | *SD* | 95% CrI | | Bayesian *R*^2^ or Δ*R*^2^ | | | |
| --- | --- | --- | --- | --- | --- | --- | --- | --- |
|  |  |  |  |  | *M* | *SD* | 95% CrI | |
|  |  |  | lower | upper |  |  | lower | upper |
| With non-neurophysiological data at t0 (*n*=28) |  |  |  |  | 0.42 | 0.07 | 0.28 | 0.55 |
| Intercept | -1.81 | 0.51 | -2.82 | -0.80 |  |  |  |  |
| Neurofeedback group (EEG, rtfNIRS) | 1.32 | 0.51 | 0.31 | 2.33 | 0.09 | 0.10 | <0.01 | 0.29 |
| Eating disorder psychopathology (EDE-Q) | -1.30 | 0.51 | -2.30 | -0.30 | 0.12 | 0.11 | <0.01 | 0.33 |
| Body mass index (kg/m^2^) | -1.69 | 0.53 | -2.73 | -0.65 | 0.16 | 0.11 | <0.01 | 0.38 |
| Perceived Stress (PSS-10)^a^ | 1.15 | 0.57 | 0.02 | 2.27 | 0.06 | 0.11 | <0.01 | 0.27 |
| With EEG data at first training session (*n*=12) |  |  |  |  | 0.25 | 0.19 | <0.01 | 0.57 |
| Intercept | -6.61 | 2.70 | -12.52 | -2.24 |  |  |  |  |
| Passive-viewing high beta power (EEG) | -4.30 | 2.17 | -8.92 | -0.50 | 0.25 | 0.19 | <0.01 | 0.57 |
| With fNIRS data at first training session (*n*=13) |  |  |  |  | 0.23 | 0.08 | 0.05 | 0.36 |
| Intercept | -0.62 | 0.48 | -1.56 | 0.31 |  |  |  |  |
| Regulation oxygenation (fNIRS)^a^ | 1.53 | 0.68 | 0.22 | 2.89 | 0.17 | 0.10 | <0.01 | 0.34 |
| Transfer oxygenation (fNIRS) | -1.69 | 0.71 | -3.12 | -0.35 | 0.18 | 0.10 | <0.01 | 0.34 |

*Notes.* CrI=credible interval, EEG=electroencephalography, fNIRS=functional near-infrared spectroscopy, EDE-Q=Eating Disorder Examination-Questionnaire (Hilbert & Tuschen-Caffier, 2016b), PSS 10=Perceived Stress Scale (Klein et al., 2016), high beta=23–28Hz, oxygenation=percentage of trials with increasing oxyhemoglobin levels (*β*>0.2), *β*=regression parameter in the applied general linear models representing the change in oxyhemoglobin levels.

^a^ 95% CrI did include 0 in the sensitivity analysis with doubled prior standard deviations.

**References**

Backhaus, J., Junghanns, K., Brocks, A., Riemann, D., & Hohagen, F. (2002). Test–retest reliability and validity of the Pittsburgh Sleep Quality Index in primary insomnia. *Journal of Psychosomatic Research, 53*, 737–740. <https://doi.org/10.1016/S0022-3999(02)00330-6>

Chatrian, G. E., Lettich, E., & Nelson, P. L. (1985). Ten percent electrode system for topographic studies of spontaneous and evoked EEG activities. *American Journal of EEG Technology, 25*, 83–92. <https://doi.org/10.1080/00029238.1985.11080163>

Delpy, D. T., Cope, M., van der Zee, P., Arridge, S., Wray, S., & Wyatt, J. (1988). Estimation of optical pathlength through tissue from direct time-of-flight measurement. *Physics in Medicine and Biology, 33*, 1433–1442. <https://doi.org/10.1088/0031-9155/33/12/008>

Grilo, C. M., & Masheb, R. M. (2007). Rapid response predicts binge eating and weight loss in binge eating disorder: Findings from a controlled trial of Orlistat with guided self-help cognitive behavioral therapy. *Behaviour Research and Therapy, 45*, 2537–2550. <https://doi.org/10.1016/j.brat.2007.05.010>

Gutzweiler, R., & In-Albon, T. (2018). Überprüfung der Gütekriterien der deutschen Version der Difficulties in Emotion Regulation Scale in einer klinischen und einer Schülerstichprobe Jugendlicher. *Zeitschrift für Klinische Psychologie und Psychotherapie, 47*, 274–286. <https://doi.org/10.1026/1616-3443/a000506>

Jerusalem, M., & Schwarzer, R. (2003). *SWE: Skala zur allgemeinen Selbstwirksamkeitserwartung* [General Self-Efficacy Scale; Test manual]. ZPID—Leibniz-Institut für Psychologie.

Klein, E. M., Brähler, E., Dreier, M., et al. (2016). The German version of the Perceived Stress Scale—Psychometric characteristics in a representative German community sample. *BMC Psychiatry, 16*, Article 159. <https://doi.org/10.1186/s12888-016-0875-9>

Lührs, M., & Goebel, R. (2017). Turbo-Satori: A neurofeedback and brain-computer interface toolbox for real-time functional near-infrared spectroscopy. *Neurophotonics, 4*, 041504. <https://doi.org/10.1117/1.NPh.4.4.041504>

Rice, M. E., & Harris, G. T. (2005). Comparing effect sizes in follow-up studies: ROC area, Cohen’s d, and r. *Law and Human Behavior, 29*, 615–620. <https://doi.org/10.1007/s10979-005-6832-7>

Robin, X., Turck, N., Hainard, A., et al. (2011). pROC: An open-source package for R and S+ to analyze and compare ROC curves. *BMC Bioinformatics, 12*, Article 77. <https://doi.org/10.1186/1471-2105-12-77>

Strobel, A., Beauducel, A., Debener, S., & Brocke, B. (2006). Eine deutschsprachige Version des BIS/BAS-Fragebogens von Carver und White. *Zeitschrift für Differentielle und Diagnostische Psychologie, 22*, 216–227. [https://doi.org/10.1024//0170-1789.22.3.216](https://doi.org/10.1024/0170-1789.22.3.216)
